# LMP2A‐Targeting CAR‐T Cells Equipped With Inducible IL‐18 to Address EBV‐Associated Malignancies

**DOI:** 10.1111/tan.70439

**Published:** 2025-10-21

**Authors:** Anna Christina Dragon, Stefanie Thoelke, Philip Mausberg, Katharina Zimmermann, Rainer Blasczyk, Michael Hudecek, Hinrich Abken, Axel Schambach, Britta Maecker‐Kolhoff, Britta Eiz‐Vesper

**Affiliations:** ^1^ Hannover Medical School Institute of Transfusion Medicine and Transplant Engineering Hannover Germany; ^2^ nextGENERATION Medical Scientist Program, Dean's Office for Academic Career Development Hannover Germany; ^3^ Institute of Experimental Hematology Hannover Medical School Hannover Germany; ^4^ Cellular Immunotherapy, Department of Internal Medicine II University of Wuerzburg Wuerzburg Germany; ^5^ Department Genetic Immunotherapy Leibniz Institute for Immunotherapy Regensburg Germany; ^6^ Genetic Immunotherapy University Regensburg Regensburg Germany; ^7^ Division of Hematology/Oncology Boston Children's Hospital Boston Massachusetts USA; ^8^ Department of Pediatric Hematology and Oncology Hannover Medical School Hannover Germany; ^9^ German Center for Infection Research (DZIF), Thematical Translation Unit‐Immunocompromised Host (TTU‐IICH) Partner Site Hannover‐Braunschweig Germany

**Keywords:** CAR‐T cells, EBV‐associated malignancies, inducible cytokine expression, LMP2A, PTLD, TCR‐like antibody, TCR‐like CAR, TRUCKs

## Abstract

Epstein–Barr virus (EBV) infects up to 95% of the world's population and persists in B cells and epithelial cells. Uncontrolled proliferation of EBV‐infected cells can result in the development of EBV‐associated malignancies, for example, post‐transplant lymphoproliferative disorder (PTLD) or nasopharyngeal cancer (NPC). It is estimated that 1.8% of deaths due to cancer worldwide are associated with EBV, and the treatment options are limited. As a new therapeutic approach, we developed chimeric antigen receptor T cells targeting EBV‐derived latent membrane protein 2A (LMP2A_CAR‐Ts). To enable specific elimination of malignant B cells infected with the intracellular pathogen, we utilised T‐cell receptor (TCR)‐like specificity to generate a CAR against the LMP2A‐derived peptide CLGGLLTMV (CLG) presented in the context of HLA‐A*02:01. To increase functionality in the tumour microenvironment, LMP2A_CAR‐Ts were additionally equipped with inducible release of IL‐12 (LMP2A_iIL‐12_TRUCKs) or IL‐18 (LMP2A_iIL‐18_TRUCKs). LMP2A_CAR‐Ts and LMP2A_iIL‐18_TRUCKs specifically recognised HLA‐A*02:01 ^+^ EBV‐transformed B‐lymphoblastoid cell lines and HLA‐A*02:01 ^+^ cells loaded with CLG peptide (A02_CLG^+^ cells), proving their target specificity, while, unexpectedly, LMP2A_iIL‐12_TRUCKs exhibited a disparate T_EM_ and NK‐like phenotype and A02_CLG‐independent reactivity. In contrast, LMP2A_CAR‐Ts and LMP2A_iIL‐18_TRUCKs effectively induced T‐cell signalling, activation, release of cytotoxic mediators, including IL‐18 by LMP2A_iIL‐18_TRUCKs and mediated cytotoxicity in a target‐specific manner. Our results demonstrate that LMP2A_CAR‐Ts and LMP2A_iIL‐18_TRUCKs specifically recognise the EBV‐derived CLG peptide presented in the context of HLA‐A*02:01. Especially, LMP2A_iIL‐18_TRUCKs with an even improved anti‐tumour response, as well as the potential to recruit bystander immune cells and overcome EBV‐mediated immune evasion strategies, might serve as a novel treatment option for various EBV‐associated malignancies.

## 1 Introduction

EBV, also known as human γ‐herpesvirus 4, infects up to 95% of the adult population and mostly establishes latency in resting memory B cells and some epithelial cells [1]. In healthy individuals, EBV reactivation is controlled by EBV‐specific T cells [2]. However, immunity impairments in patients receiving chemotherapy or immunosuppressive treatment, or in patients with autoimmune diseases, can disturb the finely tuned immune surveillance of EBV‐infected cells. As a consequence, they can proliferate and transform, resulting in malignancies [3]. A causal relationship to EBV is known for PTLD, nasopharyngeal cancer (NPC) and endemic Burkitt lymphoma (BL) [4, 5, 6]. Moreover, EBV association could be shown for more than 90% of aggressive natural killer (NK) cell leukaemia cases, 70% of diffuse large B cell lymphoma (DLBCL) cases, 70%–100% of NK/T cell lymphoma cases, 40%–80% of Hodgkin lymphoma (HL) cases, 15%–85% of non‐endemic BL cases, and up to 100% of different lymphoma cases in HIV‐positive individuals [1]. Current treatment options, including chemotherapy or depletion of all B cells, do not distinguish between the malignant EBV‐infected B cells and healthy immune cells and are thus associated with severe side effects in the already immunocompromised patients. Despite different treatment options, it is estimated that 1.8% of deaths due to cancer worldwide are associated with EBV [7].

A recent retrospective analysis evaluated the usage of CD19‐targeting CAR‐T cells (CAR‐Ts) for the treatment of 22 relapsed or refractory PTLD patients and revealed an overall response rate of 64% [8]. However, since patients with EBV‐associated malignancies are often immunocompromised, general depletion of all B cells enhances the already increased infection risk, emphasising the need for more precise targeting of malignant EBV^+^ cells in the context of EBV‐associated malignancies. Most EBV^+^ tumours display an expression pattern of EBV Latency Phase II or III, with PTLD, the most common post‐transplant malignancy, being associated with both Latency Phases II and III, and, for example, HL, DLBCL, NPC, NK cell leukaemia and NK/T cell lymphoma with EBV Latency II. The EBV protein LMP2A is expressed in these Latency Stages II and III, with a major role for EBV‐infected B‐cell persistence, viability and immune evasion by mimicking B‐cell receptor (BCR) signalling and was thus ranked as the third priority cancer antigen by a national cancer institute (NCI) pilot project [9]. Whereas it presents a promising target for specific recognition of EBV‐infected malignant cells by CAR‐Ts, LMP2A, in line with the intracellular nature of EBV, is predominantly expressed intracellularly and conventional CAR‐Ts can only target structures presented on the cell surface.

To overcome that, antigen binding domains of TCR‐like antibodies targeting specific (intracellular) target peptides in the context of distinct HLA alleles were shown to be suitable for CAR design [10, 11, 12, 13, 14, 15]. As a tool to combat EBV‐associated PTLD, we previously showed that such TCR‐like CAR‐Ts with specificity for the EBNA‐3C‐derived peptide LPPHDITPY in the context of HLA‐B*35 were suitable to eliminate EBV‐infected cells of Latency III [13]. In the current study, to target even various EBV‐associated malignancies of Latency Stages II and III and in the context of a highly prevalent HLA allele, we developed CAR‐Ts utilising the binding specificity of a previously described dimeric T‐cell engaging bispecific TCR‐like antibody targeting the LMP2A‐derived peptide CLGGLLTMV presented in the context of HLA‐A*02:01 (A02_CLG) that could mediate potent antitumor efficacy towards EBV‐transformed B lymphoblastoid cell lines in vitro and in a xenograft mouse model [12].

The success of CAR‐Ts to effectively lyse solid tumours is limited by the immunosuppressive tumour microenvironment (TME). In EBV‐associated malignancies, among other mechanisms, tumour cells release immunosuppressive soluble factors suppressing T‐cell functionality [16]. To mediate resistance towards these factors, Wagner et al. modified EBV‐specific T cells to release the cytokine IL‐12 [17]. Moreover, to oppose immune evasion strategies by EBV‐associated malignancies, the importance of NK cells was demonstrated [18, 19]. Thus, next to the A02_CLG‐targeting CAR‐Ts (LMP2A_CAR‐Ts), we here moreover generated fourth‐generation CAR‐Ts, also known as T cells redirected for universal cytokine‐mediated killing (TRUCKs) with an additional nuclear factor of activated T cells (NFAT)‐inducible expression cassette encoding for a transgenic cytokine to overcome immunosuppressive mechanisms by EBV‐associated malignancies, whereby the chemoattractive and Th1‐promoting IL‐12 (LMP2A_iIL‐12_TRUCKs) or IL‐18 (LMP2A_iIL‐18_TRUCKs), which can promote both Th1 and Th2 responses, were compared. TRUCKs induce expression of the transgenic cytokine following CAR signalling in a target‐specific and locally restricted manner, thus preventing side effects based on systemic activity [20], whereby TRUCKs with inducible release of IL‐12 or IL‐18 were demonstrated to re‐modulate the TME of solid tumours by recruitment of immune bystander cells, including M1 macrophages and NK cells [21, 22].

The results of the present study give a first evidence that LMP2A_CAR‐Ts and LMP2A_iIL‐18_TRUCKs are promising and effective new tools to treat EBV‐associated malignancies of EBV Latency II and III in a highly specific manner while maintaining the general immunity in the mostly immunocompromised patients.

## 2 Methods and Materials

### 2.1 Human Sample Materials

Peripheral blood mononuclear cells (PBMCs) were isolated from residual blood samples from routine platelet collection (Institute of Transfusion Medicine and Transplant Engineering, Hannover Medical School, Germany) by performing density gradient centrifugation with Lymphosep (c.c.pro, Oberdorla, Germany). According to standard donation requirements, the respective donors had no signs of acute infection and no previous history of blood transfusion. Written informed consent was obtained from all donors as approved by the Ethics Committee of Hannover Medical School (2519‐2014, 3639‐2017).

### 2.2 Cell Lines and Peptide Loading

All used cell lines and their cultivation medium are listed in Table S1. B‐lymphoblastoid cell lines (B‐LCLs) were generated by infecting PBMCs isolated from healthy individuals with cell culture supernatant of B95‐8 (DSMZ) as previously described [23]. Briefly, PBMCs and EBV supernatant were incubated overnight, followed by the addition of RPMI‐1640 (Lonza, Basel, Switzerland) supplemented with 20% foetal bovine serum (FBS) and 200 ng/mL cyclosporin A (both Merck, Darmstadt, Germany). As control target cells, autologous B cells were isolated (Miltenyi Biotec, Bergisch Gladbach, Germany) from PBMCs of the same individuals, cryopreserved and thawed 1 day before usage. SPI‐801 cells (ACC‐86, DSMZ, Braunschweig, Germany) are meanwhile discontinued due to a warning that they must not be used as a model for T‐ALL. In the current study, they were not used as a model for T‐ALL but as EBV negative and HLA Class I negative K‐562 (ACC‐10) subclone. Their common ancestry with K‐562 cells was confirmed using STR profiling (ATCC, Manassas, VA, USA). SPI‐801 cells were transduced with a lentiviral vector encoding HLA‐A*02:01 and sorted using PE labelled anti‐HLA‐A*02 antibodies (BioLegend, San Diego, CA, USA) and anti‐PE‐microbeads (Miltenyi Biotec) to generate SPI_A02 cells. SPI‐801 and T2 cells were transduced with HLA‐A*01:01 and sorted using FITC or PE labelled anti‐HLA‐ABC antibodies (Bio‐Rad AbD Serotec, Neuried, Germany) and either anti‐PE‐ or anti‐FITC‐microbeads (Miltenyi Biotec) or FACS sorting to receive SPI_A01 and T2_A01 cells, respectively. The HLA‐A*02:01 knockout in T2^A02ko^ and T2_A01^A02ko^ cells was received by electroporation using the Cell Line Nucleofector Kit V (Lonza, Basel, Switzerland) and the Amaxa Nucleofactor 2b (Lonza, programme A‐030) to transfer the RNP complex of 62 pmol of Alt‐R *Streptococcus pyogenes* Cas9 protein V3 precomplexed with 72 pmol duplex of HLA‐A*02:01‐targeting crRNA (5′‐ACCCAGTTCTCACTCCCATTGTTTTAGAGCTATGCT‐3′) and tracrRNA (all from Integrated DNA Technologies, Coralville, IA, USA). T2^A02ko^ and T2_A01^A02ko^ cells were then enriched using PE labelled anti‐HLA‐A*02 antibodies (BioLegend, San Diego, CA, USA) and FACS sorting.

For peptide loading, 2–5 × 10^6^ cells were cultivated in 1 mL serum‐free RPMI‐1640 (Merck) with 10 μg/mL of EBV LMP2A‐derived peptide CLGGLLTMV (CLG) or melanoma PRAME‐derived peptide SLLQHLIGL (SLL) (both Peptides & Elephants, Hennigsdorf, Germany) overnight at 37°C and 5% (v/v) CO_2_. For T2 cells, 10 μg/mL β_2_ microglobulin (Merck) was added overnight at 37°C and 5% (v/v) CO_2_.

### 2.3 Generation of LMP2A_CAR Constructs With and Without Inducible Cytokine Expression

The LMP2A_CAR was designed by synthesising (ThermoFisher Scientific, Waltham, MA, USA) the sequences of the previously reported heavy and light chain variable domains of the A02_CLG‐targeting antibody clone 38 [12]. To generate a single‐chain variable fragment (scFv), both domains were connected with a (G_4_S)_3_ linker. The scFv was cloned into the previously described TÜ165 CAR [13], for which the TÜ165 scFv was excised by using the restriction enzymes NheI‐HF and RsrII (both New England Biolabs, Ipswich, MA, USA) and replaced with the LMP2A scFv. Briefly, the LMP2A_CAR comprised the A02_CLG‐specific scFv, a 12 amino acid spacer domain derived from IgG4‐Fc (Uniprot: P01861), the cytoplasmic domain of human 4‐1BB (Uniprot: Q07011), and the cytoplasmic domain of Isoform 3 of human CD3ζ (Uniprot: P20963). Via T2A ribosomal skip element, a truncated epidermal growth factor receptor (EGFRt) sequence as selection, detection and potential depletion marker was encoded downstream of the CAR construct.

The LMP2A_CAR_iEGFP, LMP2A_CAR_iIL‐12 and LMP2A_CAR_iIL‐18 constructs for the generation of respective LMP2A_TRUCKs were generated by replacing the GD2 CAR in the previously described TRUCK vector [24] with the complete LMP2A_CAR backbone, including the EGFRt, to receive pCCL.PPT.NFATenh.synTATA.EGFP.PGK.LMP2A_CAR.T2A.EGFRt.PRE, pCCL.PPT.NFATenh.synTATA.IL12.PGK.LMP2A_CAR.T2A.EGFRt.PRE and pCCL.PPT. NFATenh.synTATA.IL18.PGK.LMP2A_CAR.T2A.EGFRt.PRE constructs. The constructs were, therefore, ‘All‐in‐One’ vectors containing both an inducible EGFP, IL‐12 (p40‐p35 single chain), or IL‐18 (matured cytokine without pro‐peptide) expression cassette, respectively, driven by an inducible promoter element with six NFAT response elements and a synthetic promoter (NFATsyn) and a constitutive PGK‐driven LMP2A_CAR expression cassette in one vector.

For cloning of the control constructs, the CD19_CAR was generated analogously to the LMP2A_CAR by replacing the TÜ165 scFv from the TÜ165‐CAR‐epHIV7 vector [13] with an FMC63‐derived scFv as previously described [25]. The scFv heavy and light chains were connected by using a Whitlow linker [26]. For the Delta_CAR, the scFv of the CD19_CAR was deleted from the construct. All other components of the CAR (spacer, 4‐1BB, CD3ζ domains) and co‐expressed EGFRt were thus the same between LMP2A_CAR, CD19_CAR and Delta_CAR constructs.

All constructs were verified by sequencing (Microsynth SeqLab, Germany). Cloning and sequence details can be provided on request.

### 2.4 Generation of Lentiviral LMP2A_CAR Vectors With and Without Inducible Cytokine Expression

Lentiviral vectors for transfer of the LMP2A_CAR, LMP2A_CAR_iEGFP, LMP2A_CAR_iIL‐12, LMP2A_CAR_iIL‐18, Delta_CAR and CD19_CAR were produced in 293 T cells. In brief, 293 T cells (DSMZ) were seeded and transfected using the calcium phosphate method and either third‐generation packaging vectors pcDNA3.HIV‐1.GP.4 × CTE (lentiviral gag/pol) [27], pRSV‐Rev (kindly provided by T. Hope, Northwestern University, Chicago, IL, USA) and VSVg‐encoding pMD.G [28] or second‐generation packaging vectors psPAX2 and pMD2.G [29]. Supernatants were harvested and concentrated via ultracentrifugation or centrifugation overnight. Titers of viral supernatants were determined by transducing Jurkat cells (DSMZ) in the presence of Polybrene Infection/Transfection Reagent (Merck). Titers were calculated based on the transduction efficiency after 48 h, which was assessed by staining with biotin‐anti‐EGFRt and PE‐Streptavidin (Thermo Fisher Scientific). Flow cytometric analysis of all samples was performed using the BD FACSCanto flow cytometer and the FlowJo_v10 Software (both Becton Dickinson, Franklin Lakes, NJ, USA).

### 2.5 Generation of LMP2A_CAR‐Ts, LMP2A_TRUCKs and Their Functional Evaluation

Untouched CD8^+^ T cells were isolated (Miltenyi) from PBMCs of healthy HLA‐A*02:01 ^*+*^ donors and were activated, transduced and expanded using a manufacturing protocol similar to the clinical manufacturing of CAR‐Ts as described [13]. Briefly, they were activated with anti‐CD3/CD28 beads (Thermo Fisher Scientific) at a 1:1 ratio and cultured in TexMACS, 3% human serum (T‐cell medium) supplemented with 12.5 ng/mL IL‐7 and IL‐15 (both from PeproTech, Cranbury, NJ, USA). After 1 day, lentiviral transduction was performed using a multiplicity of infection (MOI) of 3, the addition of Polybrene Infection/Transfection Reagent, and spinoculation. Pre‐conditioning of untransduced T cells, Delta_CAR‐Ts and LMP2A_CAR‐Ts was performed by supplementation of T‐cell medium with 10 ng/mL recombinant human IL‐12 or IL‐18/IL‐1F4 protein with carrier (both from R&D Systems, Minneapolis, MN, USA) in addition to IL‐7 and IL‐15 supplementation following lentiviral transduction. Anti‐CD3/CD28 activation beads were removed on Day 2. Enrichment of transduced cells was performed on Day 8 or 9 using anti‐EGFRt‐biotin antibody and anti‐biotin beads (Miltenyi). As a control, untransduced T cells were treated equally, except for the transduction and enrichment.

On Days 0, 4/5, 7, 8/9 and 12–15, T cells were evaluated for viability, memory and NK‐like phenotype, activation and exhaustion state using flow cytometry. All antibodies used for flow cytometry are listed in Table S2. After Days 12–15, LMP2A_CAR‐Ts and LMP2A_TRUCKs were evaluated for functionality in target cell co‐cultures. For one experiment, (transduced) T cells were frozen on Day 15 and thawed 1 day before functional evaluation. For that, 5 × 10^4^ target cells were seeded and effector cells added in an effector to target ratio (E:T ratio) of 0.5:1 or 1:1 in 200 μL T‐cell medium. After 48 h of incubation_,_ killing capacity and activation were analysed and compared between the different T cells.

### 2.6 Evaluation of LMP2A_CAR‐Induced Transcription Factor Activity Using a Reporter Assay

For investigation of target‐induced transcription factor activation by LMP2A_CAR constructs with or without inducible transgenic cytokine expression, a reporter assay based on the Jurkat T cell line JE6‐1 (kindly provided by Prof. Peter Steinberger, Medical University of Vienna, Austria) was performed as previously described [13, 30]. Briefly, JE6‐1 reporter cells were transduced with lentiviral vectors by spinoculation using an MOI of 1 and addition of Polybrene Infection/Transfection Reagent. If needed, transduced reporter cells were enriched using anti‐EGFRt‐biotin antibody and anti‐biotin beads (Miltenyi) to ensure comparable frequencies of transduced cells (Figure S1A). Target‐induced receptor signalling of transduced reporter cells was assessed by co‐culturing them with the indicated target cells in an E:T ratio of 1:1 in 200 μL RPMI 1640 (Lonza), 10% FBS and 2 mM L‐glutamine (c.c.pro). Transcription factor activity in these cells was assessed by measurement of EGFP reporting NFAT activity and enhanced cyan fluorescent protein (ECFP) reporting nuclear factor ‘kappa‐light‐chain‐enhancer’ of activated B‐cells (NF‐ΚB) activity by flow cytometry.

The specific upregulation of EGFP and ECFP in transduced JE6‐1 reporter cells following target contact was calculated by subtracting the respective fluorophore expression of reporter cells cultured alone from the respective expression in reporter cells co‐cultured with target cells.

### 2.7 Evaluation of Target‐Induced Mediator Release Using Multiplexed Cytokine Analysis

Soluble mediator concentrations in co‐culture supernatants were detected by performing a customised LEGENDplex Multi‐Analyte Flow Assay (BioLegend), allowing simultaneous quantification of human granulysin, granzyme A and B, interferon (IFN)‐γ, IL‐2, IL‐4, IL‐6, IL‐10, IL‐12(p70), IL‐18, perforin and tumour necrosis factor (TNF)‐α. For some experiments, sFasL was included as an additional analyte. Analysis was performed with the LEGENDplex Data Analysis Software Suite (BioLegend).

### 2.8 Analysis of Killing Capacity by Flow Cytometry, LDH Assay and xCELLigence

Elimination of target cells by LMP2A_CAR‐Ts and LMP2A_TRUCKs was determined using three different assays. Target‐specific killing capacity was evaluated by 7‐AAD staining (BioLegend or Becton Dickinson) and flow cytometric analysis. For that, target cells were labelled with CellTrace violet (CTV) (Thermo Fisher Scientific) before co‐culture according to the manufacturer's instructions and using an end concentration of 2 μM. Following co‐cultivation with LMP2A_CAR‐Ts and LMP2A_TRUCKs, target cell viability was measured as the frequency of 7‐AAD^−^ cells among CTV^+^ cells. Target cell viability was then normalised to the viabilities of corresponding target cells cultured alone.

For confirmation of target‐specific killing capacity, the release of lactate dehydrogenase (LDH) into the co‐culture supernatant by eliminated target cells was measured using the Cytotoxicity Detection Kit (Roche, Basel, Switzerland) according to the manufacturer's instructions and as described [13]. To obtain values for maximum lysis, one replicate per condition was treated with 1% Triton X‐100 (Merck).

For real‐time evaluation of cytotoxicity kinetics, cytotoxicity of LMP2A_CAR‐Ts and LMP2A_TRUCKs towards target cells was determined with the xCELLigence RTCA S16 Real Time Cell Analyser utilising E Plates 16 PET (both ACEA Biosciences, San Diego, CA, USA) as previously described [31]. For tethering of SPI_A02_CLG target cells, plates were coated with goat anti‐mouse IgG antibody (BioLegend) and 2 × 10^5^ cells added together with mouse anti‐human CD71 antibody (BioLegend). Background measurements were performed with RPMI 1640, 10% FBS, and 2 mM glutamine. After proof of target cell adherence to plates after 18–23 h, effector cells were added in TexMACS with 3% AB‐serum in an E:T ratio of 1:1. Impedance measurements were performed in intervals of 30 min. Cell indices were normalised to the respective indices 1 h after the addition of effector cells.

### 2.9 Statistical Analysis

Statistical analysis was conducted with GraphPad Prism V9 using two‐way ANOVA and Tukey's or Šídák's multiple comparisons tests, or Mann–Whitney tests as indicated. *p ≤ 0.05, **p ≤ 0.01, ***p ≤ 0.001, ****p ≤ 0.0001, ns > 0.05.

## 3 Results

### 3.1 LMP2A_CAR Constructs With and Without Inducible Transgenic Cytokine Expression Mediate NF‐κB and NFAT Activity Upon Recognition of A02 ^+^ EBV ^+^ or A02_CLG^+^ Target Cells

**FIGURE 1:**
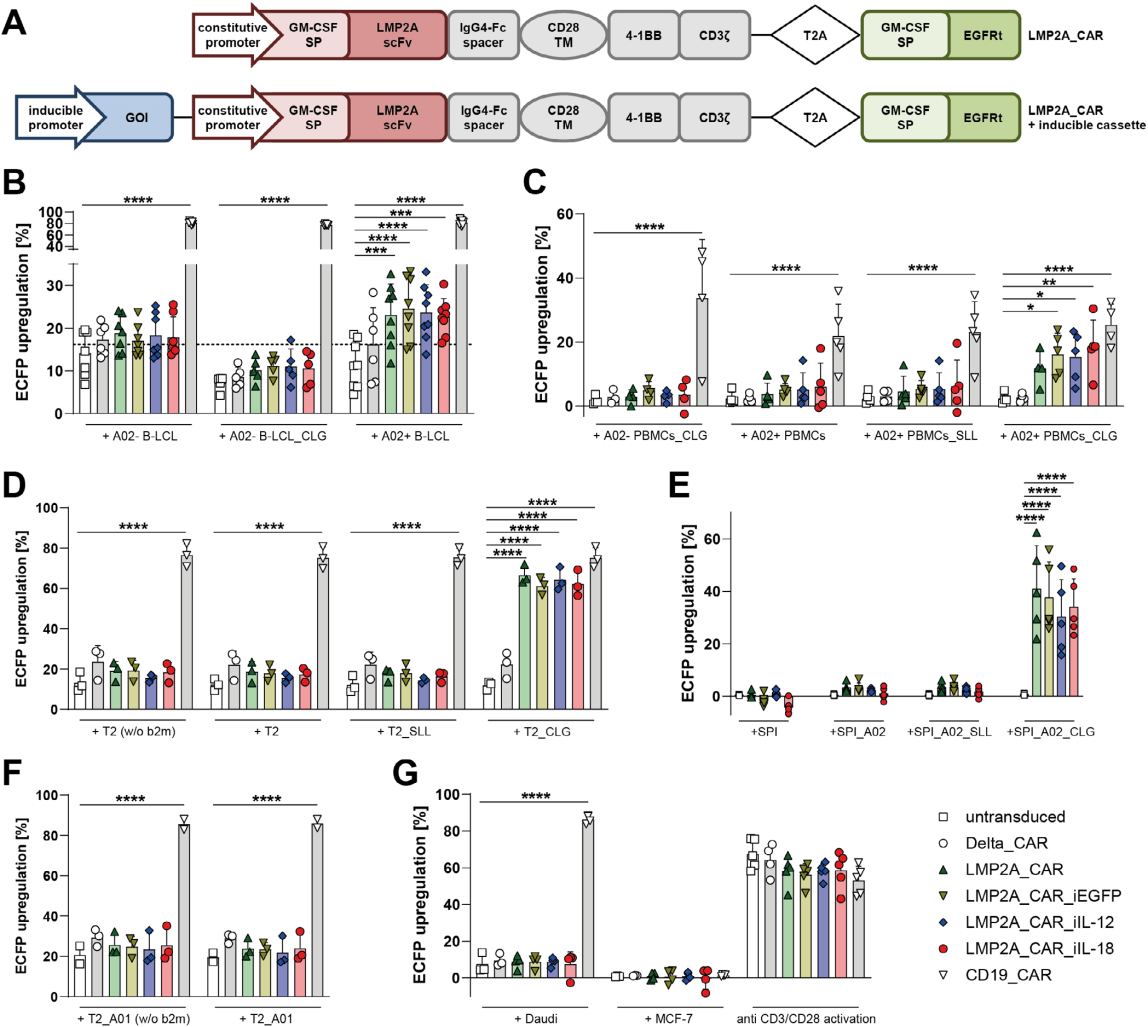
LMP2A_CAR constructs with and without inducible transgenic cytokine expression mediate NF‐κB and NFAT activity upon recognition of EBV‐infected or A02_CLG^+^ target cells. (A) All LMP2A‐targeting constructs included a constitutive promotor for the expression of the LMP2A_CAR composed of a granulocyte‐macrophage colony‐stimulating factor (GM‐CSF) signal peptide (SP), a single chain variable fragment (scFv) recognising A02_CLG, a spacer derived from IgG4‐Fc, a CD28 transmembrane domain and the cytoplasmic signalling domains of 4‐1BB and CD3ζ. The detection and selection marker truncated epidermal growth factor receptor (EGFRt) was co‐expressed by using a T2A ribosomal skip element. Constructs for LMP2A_TRUCKs additionally contained an inducible cassette composed of an NFAT‐sensitive promotor for the expression of a gene of interest (GOI), i.e., EGFP, single chain IL‐12 or IL‐18, respectively. (B‐G) The LMP2A‐targeting constructs were transduced into a JE6‐1 derived reporter cell line. A Delta_CAR construct lacking the LMP2A‐specific scFv, as well as a CD19_CAR construct with a CD19‐targeting scFv served as controls. Transduced reporter cells were co‐cultured with the indicated target cells, whereby A02‐ indicates cells derived from HLA‐A*02:01 ^*−*^ and A02+ from HLA‐A*02:01 ^*+*^ individuals, _CLG indicates cells loaded with the LMP2A‐derived peptide CLGGLLTMV, _SLL indicates cells loaded with the PRAME‐derived control peptide SLLQHLIGL, _A02 indicates cells transduced with HLA‐A*02:01 and _A01 cells transduced with HLA‐A*01:01. (D, F) To stabilise HLA expression on T2 cells, soluble β_2_ microglobulin (b2m) was added to these cells 16–24 h before co‐cultures. T2 cells without b2m addition (w/o b2m) served as controls. (G) Anti CD3/CD28 stimulation of (transduced) reporter cells served as a positive control. (B–G) After 24 h in an E:T ratio of 1:1, specific upregulation of ECFP indicating NF‐κB activity was calculated by subtracting the ECFP expression of transduced reporter cells cultured alone from the respective expression of reporter cells co‐cultured with target cells. Data are shown as mean + SD, whereby each point represents an independent experiment ((B) *n* = 5–8, (C) *n* = 3–5, (D) *n* = 3, (E) *n* = 5, (F) *n* = 2–3, (G) *n* = 3–5). Statistical analysis was performed using two‐way ANOVA and Tukey's multiple comparisons test. Only significant differences to untransduced reporter cells co‐cultured with the same target cells are shown. *p ≤ 0.05, **p ≤ 0.01, ***p ≤ 0.001, ****p ≤ 0.0001.

The A02_CLG epitope is expressed in EBV Latency Types II and III associated with several EBV‐associated malignancies. To target A02_CLG, we generated an LMP2A_CAR based on a scFv derived from a previously described A02_CLG‐specific TCR‐like antibody [12] with 4‐1BB/CD3ζ signalling domains (Figure 1A). Moreover, as an approach to overcome immunosuppressive strategies from several EBV‐associated malignancies by antagonising Th1 suppression and recruiting immune bystander cells, the constitutive expression of the LMP2A_CAR was combined with an additional NFAT‐sensitive inducible cassette encoding either IL‐12 (LMP2A_CAR_iIL‐12), IL‐18 (LMP2A_CAR_iIL‐18) or enhanced green fluorescent protein (EGFP) as a control (LMP2A_CAR_iEGFP) for the generation of corresponding LMP2A_TRUCKs (Figure 1A).

To analyse the capability of the different LMP2A‐targeting constructs to mediate specific target recognition, they were first expressed in a Jurkat JE6‐1‐derived reporter cell line indicating activation of NF‐κB by the expression of EGFP and activation of NFAT by the expression of ECFP, respectively [13, 30]. A Delta_CAR construct lacking the LMP2A‐specific scFv and a CD19_CAR construct with a CD19‐specific scFv were also transduced into reporter cells and served as controls. Expression of the selection marker EGFRt was ≥ 86% for all transduced reporter cells, with LMP2A_CAR_iEGFP and LMP2A_CAR_iIL‐18 constructs depicting slightly higher mean fluorescence intensities (MFIs; Figure S1A,B). For the LMP2A‐targeting constructs, the same high expression levels (≥ 81%) and MFI trends were observed when staining with an antibody targeting the G_4_S linker in the LMP2A‐targeting scFv (Figure S1C,D).

As target cells, PBMCs from healthy, HLA‐A*02:01 ^*−*^ (A02^−^ PBMCs) or HLA‐A*02:01 ^*+*^ individuals (A02^+^ PBMCs) were infected with EBV to generate B‐LCLs (A02^−^ B‐LCL or A02^+^ B‐LCL, respectively) known to exhibit an EBV Latency III expression profile and used as a surrogate for EBV‐associated PTLD [32]. Following co‐cultures with A02^+^ B‐LCL, all LMP2A_CAR, LMP2A_CAR_iEGFP, LMP2A_CAR_iIL‐12, LMP2A_CAR_iIL‐18 constructs significantly induced upregulation of NF‐κB activity and tended to upregulate NFAT activity, while none of these constructs responded towards A02^−^ B‐LCL, even if they were loaded with CLG peptide before (Figures 1B and S2A). Activation mediated by LMP2A_CAR constructs following co‐culture with A02^+^ B‐LCLs was even enhanced when increasing target availability on A02^+^ B‐LCLs by loading with CLG peptide (A02^+^ B‐LCL_CLG) (Figure S2B,C). As expected, the CD19_CAR induced significant transcription factor activity following co‐culture with all B‐LCLs, whereas the Delta_CAR construct did not respond to either of them, confirming that target recognition of A02^+^EBV^+^ cells by LMP2A_CAR constructs was mediated by their LMP2A‐specific scFv. To prove EBV specificity of LMP2A_CAR activation, the transduced reporter cells were co‐cultured with uninfected A02^+^ B cells and A02^+^ PBMCs (Figures 1C and S2B–D). In neither of these co‐cultures did LMP2A_CAR constructs induce NF‐κB or NFAT activity and was only measured when A02^+^ PBMCs were loaded with CLG peptide, but not a melanoma‐derived control peptide SLL, before.

To further analyse the specificity of the different LMP2A_CAR constructs for the A02_CLG epitope in detail, EBV‐negative T2 and SPI‐801 (SPI) cells were utilised as additional target cell models. T2 cells express HLA‐A*02:01 with the same expression level as A02^+^ PBMCs, whereas SPI cells are HLA Class I negative and were transduced to express HLA‐A*02:01 (SPI_A02) to a similar HLA expression level (Figure S1E,F). None of the LMP2A_CAR construct transduced reporter cells showed NF‐κB or NFAT activity in co‐cultures with the control cells T2, T2 cells loaded with the SLL control peptide (T2_SLL), SPI, SPI_A02, or SPI_A02 loaded with SLL peptide (SPI_A02_SLL; Figures 1D,E and S2E,F). In contrast, in all LMP2A_CAR‐transduced reporter cells, NF‐κB and NFAT activity were significantly induced in co‐cultures with T2 or SPI_A02 cells that were CLG‐loaded (T2_CLG and SPI_A02_CLG) in comparison to corresponding co‐cultures of untransduced reporter cells. To confirm HLA specificity, T2 and SPI cells were transduced to express HLA‐A*01:01 (T2_A01 and SPI_A01), towards which none of the LMP2A_CAR constructs reacted, even not if SPI_A01 were loaded with CLG peptide (SPI_A01_CLG; Figures 1F and S2G–I).

As negative controls, HLA‐negative Daudi cells and HLA‐A*02 ^+^ MCF‐7 cells, which are EBV^−^ but are described to express a peptide similar to CLG [12], were used as target cells. Neither of the LMP2A_CAR constructs induced NF‐κB or NFAT activity following co‐culture with these cells (Figures 1G and S2J).

Thus, all generated LMP2A_CAR constructs, with or without inducible transgenic cytokine expression, efficiently induced NFAT and NF‐κB activity in specific response to various A02^+^EBV^+^ or A02_CLG^+^ target cells.

### 3.2 LMP2A_CAR‐Ts, LMP2A_iEGFP_TRUCKs and LMP2A_iIL‐18_TRUCKs Have a Favourable Phenotype With a Low Level of Activation and Exhaustion

**FIGURE 2:**
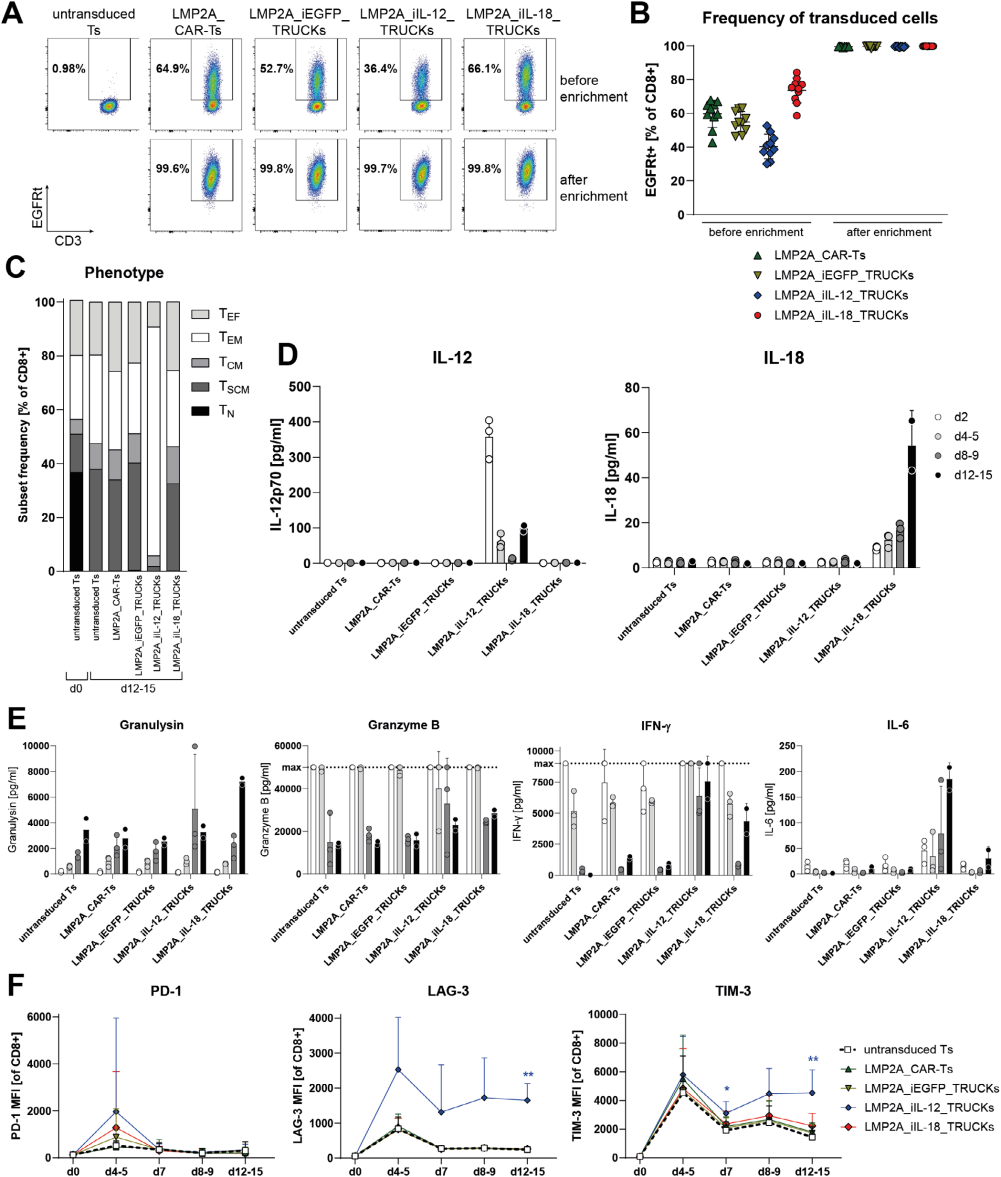
Generated LMP2A_CAR‐Ts, LMP2A_iEGFP_TRUCKs and LMP2A_iIL‐18_TRUCKs have a favourable phenotype with a low level of activation and exhaustion. The LMP2A‐targeting constructs were transduced into CD8^+^ T cells isolated from healthy individuals. Respective untransduced T cells (untransduced Ts) served as controls. (A, B) The frequency of transduced cells was determined on Days 8–9 by staining with biotin‐anti‐EGFRt and PE‐streptavidin before and after the enrichment of transduced cells. Data are shown as (A) representative plots and (B) mean ± SD, whereby each point represents one donor (*n* = 10). (C) The phenotype of LMP2A_CAR‐Ts and TRUCKs during expansion was assessed on the indicated days as naïve (T_N_: CD45RO^−^ CCR7^+^ CD95^−^), stem‐cell memory (T_SCM_: CD45RO^−^ CCR7^+^ CD95^+^), central memory (T_CM_: CD45RO^+^ CCR7^+^), effector memory (T_EM_: CD45RO^+^ CCR7^−^) and effector (T_EF_: CD45RO^−^ CCR7^−^) T cells. Data are shown as mean (*n* = 4). (D, E) By performing a bead‐based multiplex cytokine profiling using flow cytometry, the release of the indicated soluble mediators was detected in the cell culture supernatant during expansion. Data are shown as mean + SD, whereby each point represents one donor (*n* = 2–3). (F) The indicated markers for exhaustion were analysed during expansion using flow cytometry. Data are shown as mean + SD (*n* = 7–8). Statistical analysis was performed using two‐way ANOVA and Tukey's multiple comparisons test. Significant differences to values obtained for untransduced Ts on the same day are indicated. *p ≤ 0.05, **p ≤ 0.01. MFI = mean fluorescence intensity.

The LMP2A_CAR constructs were then transduced into CD8^+^ T cells isolated from healthy individuals, whereby untransduced T cells served as controls. Using an expansion protocol similar to the clinical manufacturing of CAR‐Ts [13], 42‐ to 70‐fold‐expansion of transduced T cells was achieved within 8–9 days (Figure S3A). The frequency of transduced cells was 60% for LMP2A_CAR‐Ts, 55% for LMP2A_iEGFP_TRUCKs, 40% for LMP2A_iIL‐12_TRUCKs, and 74% for LMP2A_iIL‐18_TRUCKs, respectively (Figure 2A,B). They were enriched by a co‐expressed EGFRt as a detection and selection marker, resulting in the purity for all engineered T cells to at least 99% EGFRt^+^ cells, and CAR expression levels were confirmed to be ≥ 82% using G_4_S staining (Figure S3B). After enrichment, LMP2A_CAR‐Ts and TRUCKs could be further expanded three‐ to sixfold until Days 12–15 (Figure S3A). The memory phenotype of LMP2A_CAR‐Ts, LMP2A_iEGFP_TRUCKs and LMP2A_iIL‐18_TRUCKs on Days 12–15 was, similar to untransduced T cells, mainly stem‐cell‐like memory T cells (T_SCM_; 32%–38%) and effector memory T cells (T_EM_; 26%–33%; Figures 2C and S3C). In contrast, LMP2A_iIL‐12_TRUCKs shifted primarily to T_EM_ cells (85%) with almost absent T_SCM_ cells (1.8%). To determine whether this was due to the release of inducible cytokines by the engineered T cells, concentrations of IL‐12 and IL‐18 in the cell culture supernatants during cultivation were assessed (Figure 2D). IL‐12 was released by LMP2A_iIL‐12_TRUCKs during the whole expansion with the highest levels after 2 days (mean of 360 pg/mL), most likely resulting from the stimulation with anti‐CD3/CD28 beads inducing NFAT activity at the beginning of T‐cell generation. However, IL‐12 concentrations in the supernatant decreased until Days 13–15 of the generation of LMP2A_iIL‐12_TRUCKs. LMP2A_iIL‐18_TRUCKs showed a markedly lower IL‐18 release during generation with the highest mean value of 54 pg/mL at the end of cultivation. As expected, untransduced T cells as well as LMP2A_CAR‐Ts and LMP2A_iEGFP_TRUCKs did not release IL‐12 or IL‐18 during expansion.

To further evaluate the influence of the released IL‐12 and IL‐18 by respective LMP2A_TRUCKs, secretion of pro‐inflammatory mediators, as well as exhaustion and activation states of the different LMP2A‐targeting T cells was evaluated during their generation (Figures 2E,F and S3D–K). Along with the initial anti‐CD3/CD28 treatment, untransduced T cells, LMP2A_CAR‐Ts, and TRUCKs were activated, indicated by an increased expression of CD25, CD137, and FasL on Days 4–5, which mainly decreased again until Days 12–15 (Figure S3D–F). In line, all LMP2A‐targeting T cells released pro‐inflammatory mediators during generation, whereby LMP2A_iIL‐12_TRUCKs released more IFN‐γ and IL‐6 towards the end of generation and LMP2A_iIL‐18_TRUCKs more IFN‐γ when compared to untransduced T cells and LMP2A_CAR‐Ts (Figure 2E). Remarkably, whereas untransduced T cells, LMP2A_CAR‐Ts, LMP2A_iEGFP_TRUCKs, and LMP2A_iIL‐18_TRUCKs depicted a similar pattern of exhaustion marker PD‐1, LAG‐3, TIM‐3, 2B4 and CD39 expression during expansion with only less indication for exhaustion after 12–15 days of expansion, LMP2A_iIL‐12_TRUCKs displayed similarly low levels of PD‐1 and 2B4, but a significantly increased expression of LAG‐3 and TIM‐3, and a slightly elevated expression of CD39 (Figures 2F and S3G–K).

Thus, the generated LMP2A_CAR‐Ts, LMP2A_iEGFP_TRUCKs, and LMP2A_iIL‐18_TRUCKs showed a favourable phenotype with low levels of activation and exhaustion. Whereas low levels of IL‐18 released by LMP2A_iIL‐18_TRUCKs during generation did not alter their memory phenotype, activation, and exhaustion levels, LMP2A_iIL‐12_TRUCKs, most likely due to anti‐CD3/CD28 stimulation‐mediated IL‐12 release, showed a higher level of differentiation, release of pro‐inflammatory mediators, and expression of markers associated with exhaustion.

### 3.3 LMP2A_TRUCKs Specifically Activate the Inducible Cassette After Target Recognition

**FIGURE 3:**
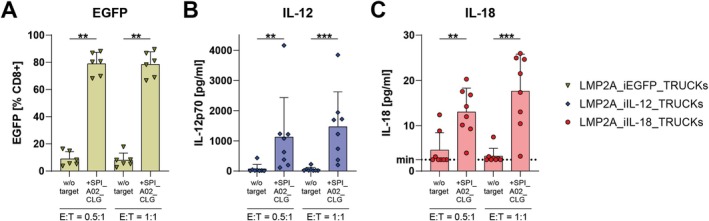
LMP2A_TRUCKs specifically activate the inducible cassette after target recognition. LMP2A_TRUCKs were either cultured alone (w/o target) or co‐cultured with HLA‐A*02:01^+^ SPI‐801 cells loaded with CLG peptide (SPI_A02_CLG) in the indicated effector‐to‐target (E:T) ratios for 48 h. (A) EGFP expression of LMP2A_iEGFP_TRUCKs was determined by flow cytometry (*n* = 6). By performing a bead‐based multiplex cytokine profiling using flow cytometry, the release of (B) IL‐12 by LMP2A_iIL‐12_TRUCKs or (C) IL‐18 by LMP2A_iIL‐18_TRUCKs was analysed in the respective culture supernatants (*n* = 8). The lower limit of detection (min) is indicated on the *y*‐axis. (A–C) Data are shown as mean + SD, whereby each point represents one donor. Statistical analysis was performed using the Mann–Whitney test. **p ≤ 0.01, ***p ≤ 0.001.

After their generation, the LMP2A_TRUCKs were evaluated for activation of their respective inducible cassettes following target‐induced receptor signalling using SPI_A02_CLG cells in different E:T ratios (Figure 3). LMP2A_iEGFP_TRUCKs showed significantly enhanced expression of EGFP after co‐culture with SPI_A02_CLG cells when compared to respective LMP2A_iEGFP_TRUCKs cultured alone (Figure 3A). Accordingly, concentrations of IL‐12 and IL‐18 in the co‐culture supernatants of LMP2A_iIL‐12_TRUCKs or LMP2A_iIL‐18_TRUCKs, respectively, were measured by bead‐based multiplex cytokine profiling and shown to be significantly increased following co‐culture with SPI_A02_CLG compared to respective LMP2A_TRUCKs cultured alone (Figure 3B,C). While the IL‐12 released by LMP2A_iIL‐12_TRUCKs reached a mean concentration of above 1100 pg/mL, the IL‐18 concentration released by LMP2A_iIL‐18_TRUCKs raised to mean levels of 18 pg/mL in an E:T ratio of 1:1.

Taken together, the inducible cassettes in all LMP2A_TRUCKs were activated following CAR activation upon target cell contact.

### 3.4 LMP2A_CAR‐Ts and LMP2A_iIL‐18_TRUCKs Are Specifically Activated and Release Pro‐Inflammatory Mediators Following Recognition of A02_CLG^+^ Target Cells

**FIGURE 4:**
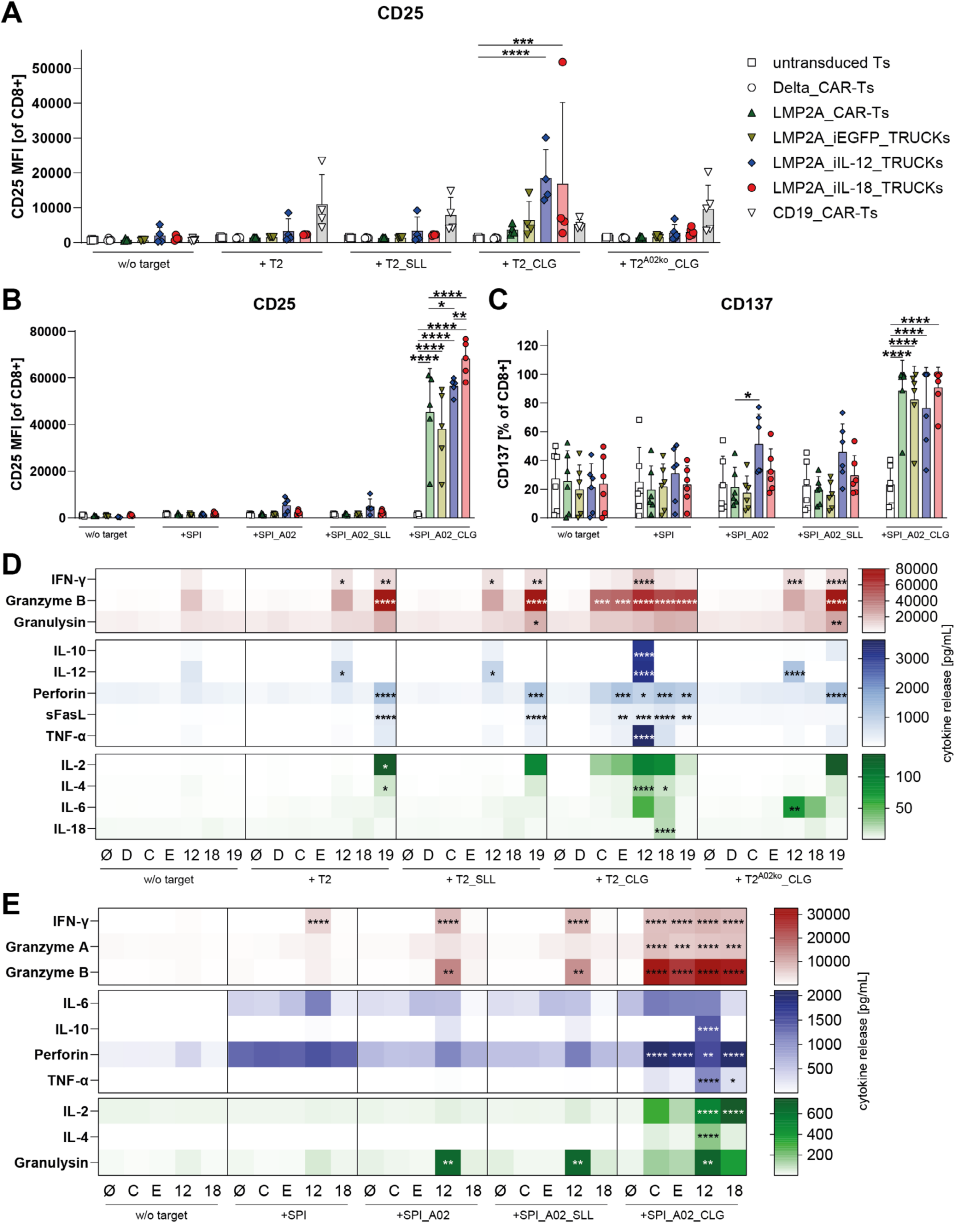
LMP2A_CAR‐Ts and LMP2A_TRUCKs are specifically activated and release pro‐inflammatory mediators following recognition of A02_CLG^+^ target cells. LMP2A_CAR‐Ts and LMP2A_TRUCKs were either cultured alone (w/o target) or co‐cultured with (A, D) T2 cells, T2 cells loaded with the LMP2A‐derived peptide CLGGLLTMV (T2_CLG), T2 cells loaded with the PRAME‐derived control peptide SLLQHLIGL (T2_SLL) or CLG‐loaded T2 cells with *HLA‐* A*02:01 knockout (T2^A02ko^_CLG) for 48 h in an E:T ratio of 1:1 or (B, C, E) untransduced SPI‐801 cells (SPI), SPI transduced with HLA‐A*02:01 either unloaded (SPI_A02), CLG‐loaded (SPI_A02_CLG) or SLL‐loaded (SPI_A02_SLL) for 48 h in an E:T ratio of 0.5:1. (A, D) Corresponding co‐cultures with Delta_CAR‐Ts lacking the LMP2A‐specific scFv or CD19_CAR‐Ts with a CD19‐targeting scFv served as controls. (A–C) Markers for T‐cell activation were determined by flow cytometry and are shown as (A, B) mean fluorescence intensity (MFI) or (C) frequency of positive cells. (D, E) The concentration of different mediators in the culture supernatants was analysed by a bead‐based multiplex cytokine profiling using flow cytometry. Significant differences to values obtained for untransduced Ts co‐cultured with the same target cells are indicated. 12 = LMP2A_iIL‐12_TRUCKs, 18 = LMP2A_iIL‐18_TRUCKs, 19 = CD19_CAR‐Ts, C = LMP2A_CAR‐Ts, D = Delta_CAR‐Ts, E = LMP2A_iEGFP_TRUCKs, ø = untransduced Ts. Data are shown as (A–C) mean + SD or (D, E) mean, whereby each point represents one individual experiment ((A, D) *n* = 3–5 with T cells from *n* = 3–4 donors, (B, C) *n* = 4–6 donors, (E) *n* = 6–8 donors). Statistical analysis was performed using two‐way ANOVA and Tukey's multiple comparisons test. Only significant differences to untransduced T cells co‐cultured with the same target cells are shown. *p ≤ 0.05, **p ≤ 0.01, ***p ≤ 0.001, ****p ≤ 0.0001.

Functionality of LMP2A_CAR‐Ts and LMP2A_TRUCKs was evaluated by their activation response and release of pro‐inflammatory mediators following co‐culture with T2_CLG or SPI_A02_CLG. (Figures 4, S4 and S5). Following co‐culture with T2_CLG cells, expression of activation marker CD137 was significantly increased on all LMP2A‐targeting T cells and CD25 expression was significantly increased on both LMP2A_iIL‐12_TRUCKs and LMP2A_iIL‐18_TRUCKs when compared to untransduced T cells cultured with T2_CLG (Figure 4A and S4A). Following co‐culture with SPI_A02_CLG cells, the expression of activation marker CD25 and CD137, as well as FasL as a mediator of target cell elimination, was significantly increased on all LMP2A‐targeting T cells (Figures 4B,C and S5A–D). In that, LMP2A_iIL‐18_TRUCKs induced expression of CD25 significantly more when compared with the other LMP2A‐targeting T cells. In contrast, LMP2A_CAR‐Ts, LMP2A_iEGFP_TRUCKs and LMP2A_iIL‐18_TRUCKs did not respond to all control cells, including T2, T2_SLL, T2_A01, SPI, SPI_A02, SPI_A02_SLL, SPI_A01 and SPI_A01_CLG (Figures 4A–C, S4 and S5). To evaluate HLA specificity in co‐cultures with T2 cells, HLA‐A*02:01 was knocked out in T2 (T2^A02ko^) and T2_A01 cells (T2_A01^A02ko^) using CRISPR/Cas9 (Figure S1F). Neither of the LMP2A_CAR‐Ts nor LMP2A_TRUCKs significantly upregulated activation marker expression following co‐culture with CLG‐loaded T2^A02ko^ (T2^A02ko^_CLG) or CLG‐loaded T2_A01 cells (T2_A01^A02ko^_CLG, Figures 4A and S4). As the only response that was not restricted to A02_CLG^+^ target recognition, LMP2A_iIL‐12_TRUCKs significantly upregulated CD137 and Fas‐L expression following co‐culture with SPI_A02, as well as CD25 and CD137 expression following co‐culture with SPI_A01 cells (Figures 4C and S5B,D–F).

In addition, LMP2A_CAR‐Ts and TRUCKs released pro‐inflammatory cytokines and cytotoxic mediators subsequent to A02_CLG target recognition (Figures 4D,E and S6A,B). This included significant upregulation of granzyme B, IFN‐γ, granzyme A and perforin by all LMP2A_CAR‐Ts and TRUCKs, significantly increased release of IL‐18 and IL‐2 by LMP2A_iIL‐18_TRUCKs and significantly higher secretion of IL‐4, IL‐10, IL‐12 and TNF‐α by LMP2A_iIL‐12_TRUCKs when they were co‐cultured with T2_CLG or SPI_A02_CLG. Whereas LMP2A_CAR‐Ts, LMP2A_iEGFP_TRUCKs and LMP2A_iIL‐18_TRUCKs did not release more of any of the tested pro‐inflammatory mediators in co‐cultures with the control cells (T2, T2_SLL, T2^A02ko^_CLG, T2_A01, T2_A01^A02ko^_CLG, SPI, SPI_A02 or SPI_A02_SLL), LMP2A_iIL‐12_TRUCKs exhibited a significantly increased release of IFN‐γ, IL‐12 and granzyme B after co‐culture with most of the mentioned control cells irrespective of the presence of CLG.

Taken together, LMP2A_CAR‐Ts and LMP2A_iIL‐18_TRUCKs are specifically activated and release pro‐inflammatory mediators upon recognition of the A02_CLG epitope. The LMP2A_iIL‐12_TRUCKs exhibit a target‐antigen independent response towards control cells regarding expression of CD137 and Fas‐L, as well as the release of IFN‐γ, IL‐12, and granzyme B.

### 3.5 LMP2A_CAR‐Ts and LMP2A_iIL‐18_TRUCKs Eliminate A02_CLG^+^ Target Cells With High Specificity, Whereas LMP2A_iIL‐12_TRUCKs Also Eliminate A01^+^ and A02 ^+^ Target Cells

**FIGURE 5:**
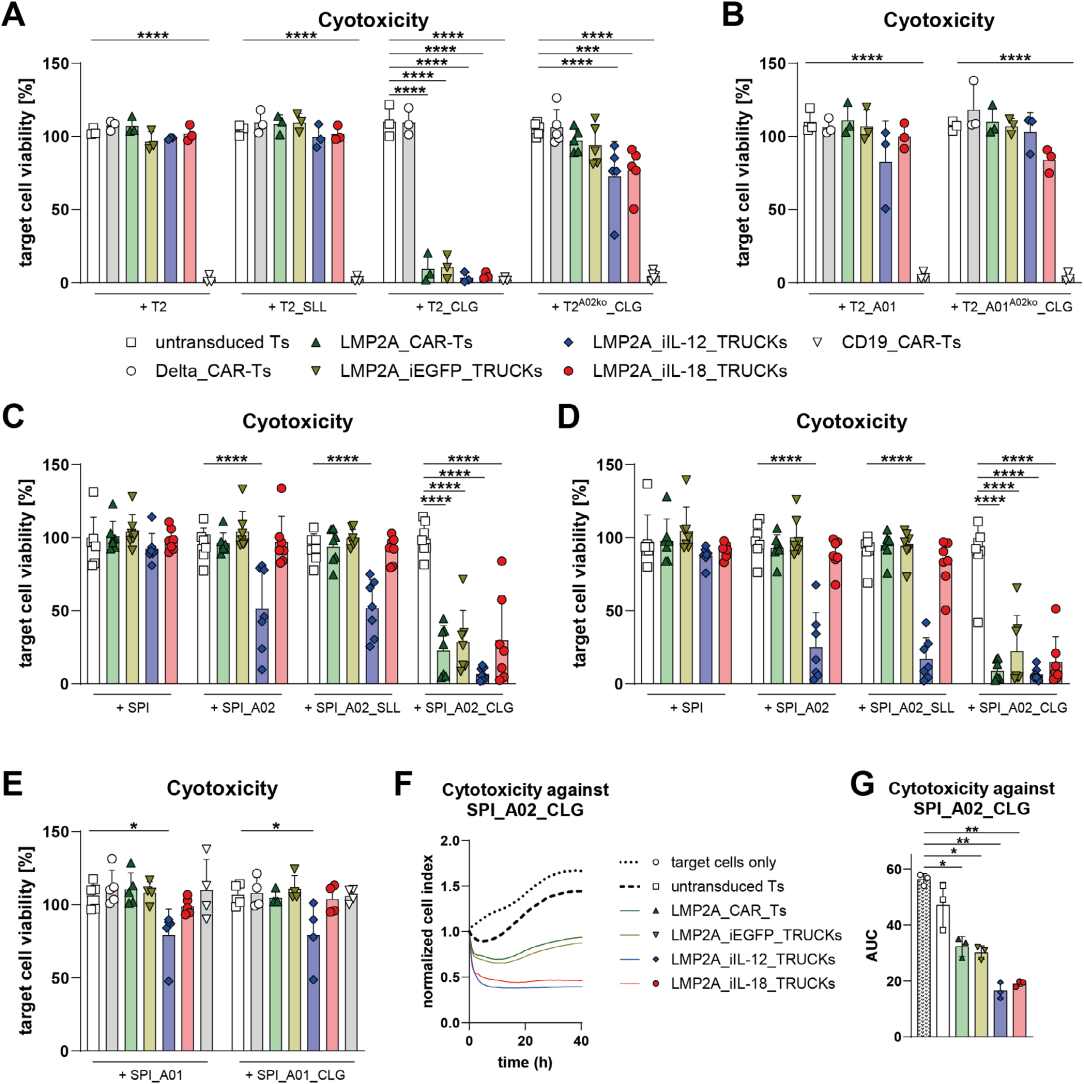
LMP2A_CAR‐Ts and LMP2A_iIL‐18_TRUCKs eliminate A02_CLG^+^ target cells with high specificity, whereas LMP2A_iIL‐12_TRUCKs also eliminate A01^+^ and A02^+^ target cells. LMP2A_CAR‐Ts and LMP2A_TRUCKs were either cultured alone (w/o target) or co‐cultured with (A, B) T2 cells, T2 cells loaded with the LMP2A‐derived peptide CLGGLLTMV (T2_CLG), T2 cells loaded with the PRAME‐derived control peptide SLLQHLIGL (T2_SLL), CLG‐loaded T2 cells with HLA‐A*02:01 knockout (T2^A02ko^_CLG), T2 cells transduced with HLA‐A*01:01 (T2_A01) or CLG‐loaded T2_A01 with HLA‐A*02:01 knockout (T2_A01^A02ko^_CLG) for 48 h in an E:T ratio of 1:1 or (C–E) untransduced SPI‐801 cells (SPI), SPI transduced with HLA‐A*02:01 either unloaded (SPI_A02), CLG‐loaded (SPI_A02_CLG) or SLL‐loaded (SPI_A02_SLL), SPI transduced with HLA‐A*01:01 (SPI_A01) or CLG‐loaded SPI_A01 (SPI_A01_CLG) for 48 h in an E:T ratio of (C) 0.5:1 or (D–G) 1:1. (A, B, E) Corresponding co‐cultures with Delta_CAR‐Ts lacking the LMP2A‐specific scFv or CD19_CAR‐Ts with a CD19‐targeting scFv served as controls. (A–E) The cytotoxic activity of LMP2A_CAR‐Ts and LMP2A_TRUCKs was determined by analysing the viability of CTV‐labelled target cells by 7‐AAD staining and subsequent flow cytometry analysis. Target cell viability was normalised to viabilities of corresponding target cells cultured alone ((A, B, E) *n* = 3–5 with T cells from *n* = 3–4 donors, (C, D) *n* = 7 donors). (F, G) Cytotoxicity against SPI_02_CLG was analysed in detail by real‐time impedance measurements every 30 min using the xCELLigence RTCA S16 Real Time Cell Analyser. Data are normalised to the respective cell indices at the time point of T‐cell addition for each condition. Normalised cell indices are shown as (F) mean or (G) calculated area under the curve (AUC) values (*n* = 3). (A–E, G) Data are shown as mean + SD, whereby each point represents one donor. Statistical analysis was performed using two‐way ANOVA and Tukey's multiple comparisons test. Only significant differences to untransduced T cells co‐cultured with the same target cells are shown. *p ≤ 0.05, **p ≤ 0.01, ***p ≤ 0.001, ****p ≤ 0.0001.

To assess the cytotoxic potential of LMP2A_CAR‐Ts and LMP2A_TRUCKs, the viability of CTV‐labelled target cells following co‐culture with the different LMP2A‐targeting T‐cells was determined after 48 h by 7‐AAD staining (Figure 5A–E and S5C). LMP2A_CAR‐Ts and TRUCKs did not significantly affect the viability of T2, T2_SLL, T2_A01, T2_A01^A02ko^_CLG and SPI cells. Moreover, neither LMP2A_CAR‐Ts nor LMP2A_iEGFP_TRUCKs or LMP2A_iIL‐18_TRUCKs reduced the viability of the control cells SPI_A02, SPI_A02_SLL, SPI_A01 and SPI_A01_CLG. In contrast, all LMP2A_CAR‐Ts and TRUCKs induced a significant decrease in T2_CLG viability below 3.3%–10% that was similar to the viability reduction mediated by CD19_CAR‐Ts (2.3%; Figure 5A). In line, they reduced the viability of SPI_A02_CLG cells below 30% in an E:T ratio of 0.5:1 and below 22% in an E:T ratio of 1:1. T2^A02ko^_CLG viability was slightly but significantly reduced by LMP2A_iIL‐12_TRUCKs and LMP2A_iIL‐18_TRUCKs, most likely due to residual HLA‐A*02 ^+^ cells that were CLG‐loaded. In co‐cultures with T2_CLG and SPI_A02_CLG cells, LMP2A_iIL‐12_TRUCKs mediated the highest cytotoxicity when compared to the other engineered T cells, but also led to the elimination of SPI_A02, SPI_A02_SLL, SPI_A01, and SPI_A01_CLG cells (viability 17%–79%), but not SPI cells (Figure 5A,C–E). In line, the viability of T2_A01 cells tended to be reduced in co‐cultures with LMP2A_iIL‐12_TRUCKs (Figure 5B). This indicates an off‐target cytotoxic effect directed towards transduced HLA molecules on SPI‐801 (and T2) cells irrespective of the presence of the A02_CLG epitope.

Cytotoxicity by LMP2A_CAR‐Ts and TRUCKs was further confirmed by determination of the LDH release into the supernatant of the same co‐cultures with an E:T ratio of 1:1 and revealed that LMP2A_CAR‐Ts and LMP2A_iIL‐18_TRUCKs exhibited significant cytotoxicity against SPI_A02_CLG, but not towards SPI, SPI_A02 and SPI_A02_SLL cells (Figure S6D). However, again a significant off‐target cytotoxicity of LMP2A_iIL‐12_TRUCKs towards HLA‐A*02:01 present on SPI_A02 and SPI_A02_SLL was confirmed.

To investigate further on the A02_CLG independent reactivity of LMP2A_iIL‐12_TRUCKs, the influence of IL‐12 pre‐conditioning, in comparison to IL‐18 pre‐conditioning, on the phenotype and target response of (transduced) T cells was evaluated (Figures S7 and S8). Following the addition of IL‐12, but not IL‐18 in the same concentration, to untransduced T cells, Delta_CAR‐Ts of LMP2A_CAR‐Ts during their generation, a memory phenotype of primarily T_EM_ cells was induced that was similar to the phenotype of LMP2A_iIL‐12_TRUCKs (Figure S7A,E). Moreover, IL‐12 pre‐conditioned Delta_CAR‐Ts (IL‐12_Delta_CAR‐Ts) and LMP2A_CAR‐Ts (IL‐12_LMP2A_CAR‐Ts), similar to LMP2A_iIL‐12_TRUCKs, depicted a significantly lower expansion and cell viability towards the end of their generation (Day 15; Figure S7B,C). Interestingly, these cells exhibited a significant proportion of CD56^+^CD94^+^CD62L^+^ cells (13%–16%) that are described to exhibit an NK‐like phenotype [33] (Figure S7D). LMP2A_iIL‐12_TRUCKs exhibited a similar frequency (15%) of NK‐like cells after their generation, whereas none of the other untransduced or transduced T cells, also not IL‐18 pre‐conditioned cells, showed similarly altered memory phenotype, expansion and viability as well as NK phenotype (Figure S7D–H).

The pre‐conditioned T cells were then co‐cultured with SPI_A01 and SPI_A02, as well as HLA‐negative Daudi and HLA‐A*02 ^*+*^ *EBV* ^*−*^ MCF‐7 cells (Figure S8). Similar to LMP2A_iIL‐12_TRUCKs, IL‐12_Delta_CAR‐Ts and IL‐12_LMP2A_CAR‐Ts tended to increase activation marker CD25 and CD137 expression, significantly upregulated release of several pro‐inflammatory mediators (IFN‐γ, IL‐6, granzyme B, perforin, sFasL and IL‐2) and slightly increased the secretion of further cytokines following co‐cultures with SPI_A01, SPI_A02, or MCF‐7 cells (Figure S8A–C). The viability of SPI_A01 and SPI_A02 was slightly reduced, and the viability of MCF‐7 cells significantly reduced following co‐culture with IL‐12_Delta_CAR‐Ts, IL‐12_LMP2A_CAR‐Ts, and LMP2A_iIL‐12_TRUCKs (Figure S8D). Only the HLA‐negative Daudi cells were not eliminated by any of the (pre‐conditioned) T cells.

Thus, A02_CLG independent reactivity of LMP2A_iIL‐12_TRUCKs was most likely not mediated by the LMP2A‐specific scFv, but IL‐12 mediated, as IL‐12 pre‐conditioning of transduced cells induced the same phenotype and target response.

### 3.6 LMP2A_iIL‐18_TRUCKs Mediate More Immediate Elimination of A02_CLG^+^ Target Cells Compared to LMP2A_CAR‐Ts Indicated by Real‐Time Analysis

For a more detailed comparison of the kinetics of A02_CLG‐specific cytotoxicity mediated by the different LMP2A‐targeting T cells, real‐time measurements using an xCELLigence Real Time Cell Analyser were performed (Figure 5F,G). Impedance measurements every 30 min allowed evaluation of the detachment of SPI_A02_CLG cells in co‐cultures with LMP2A_CAR‐Ts or LMP2A_TRUCK as a surrogate for induced target‐cell death. SPI_A02_CLG cells cultured alone or with untransduced T cells expanded, as shown by increasing normalised cell indices with mean values of 1.4–1.7 after 40 h (Figure 5F). Strikingly, all LMP2A_CAR‐Ts and TRUCKs reduced the viability of SPI‐A02_CLG cells, whereby LMP2A_iIL‐12_TRUCKs and LMP2A_iIL‐18_TRUCKs showed markedly increased and more immediate cytotoxicity. The mean normalised cell indices of SPI‐A02_CLG in these co‐cultures were 0.4 and 0.5, respectively, already after 5 h of co‐cultivation and remained stable until the endpoint of the experiment after 40 h. LMP2A_CAR‐Ts and LMP2A_iEGFP_TRUCKs, after the initial response (mean indices of 0.7 after 5 h), permitted partial regrowth of SPI‐A02_CLG cells (mean indices of 0.9 after 40 h). Quantification of area under the curve (AUC) values revealed the significance of cytotoxicity mediated by all LMP2A‐targeting T cells (Figure 5G).

Thus, A02_CLG epitope‐specific and efficient cytotoxicity of LMP2A_CAR‐Ts and LMP2A_iIL‐18_TRUCKs was demonstrated by independent methods.

## 4 Discussion/Conclusions

The LMP2A‐derived A02_CLG epitope presents a highly specific and promising therapeutic target as it is expressed in EBV Latency Type III and II associated with several EBV‐associated malignancies. These include EBV‐associated PTLD, NPC, NK cell leukaemia, and NK/T cell lymphoma, DLBCL and HL, for all of which current standard treatment options do not induce specific elimination of malignant EBV‐infected cells and are thus associated with severe side‐effects [1]. As a specific cellular therapy approach for EBV‐associated PTLD, EBV‐specific memory T cells directly isolated or expanded from the blood of an EBV‐seropositive donor can be adoptively transferred to restore EBV‐specific T‐cell immunity and could be shown to control malignant cell proliferation in patients [32, 34, 35, 36]. However, their use is limited by the need for an appropriate, at least partially HLA‐matched donor with sufficient EBV‐specific T‐cell numbers. To circumvent the need for a suitable allogeneic donor and broaden applicability, we here developed TCR‐like LMP2A_CAR‐Ts and TRUCKs targeting A02_CLG by utilising the binding specificity of a previously described A02_CLG‐specific TCR‐like antibody [12] for the potential use as a novel cell‐therapy approach.

Capability of LMP2A_CAR constructs with or without inducible transgenic cytokine expression cassettes to recognise EBV‐infected and ‐transformed B cells could be demonstrated by NF‐κB and NFAT activation following contact with EBV‐infected B‐LCLs from HLA‐A*02:01 ^*+*^, but not HLA‐A*02:01 ^*−*^ individuals. In contrast, none of the LMP2A_CAR constructs mediated transcription factor activity against EBV‐uninfected B cells or PBMCs. Thus, CAR activity was restricted to A02^+^EBV^+^ cells. Due to their EBV latency III profile, B‐LCLs are widely used as surrogates for EBV‐associated PTLD and are also used for the manufacturing of EBV‐specific T cells for clinical application to PTLD patients by several groups [32, 34, 36]. Whereas the current study focused further on dissecting HLA and peptide specificity of LMP2A_CAR‐Ts and LMP2A_TRUCKs by using various T2, SPI, Daudi and MCF‐7 cells, their anti‐tumour efficacy towards EBV‐infected cells should be investigated more in detail in future experiments, including in vivo evaluation. This would also allow comparability with the A02_CLG‐targeting antibody clone 38 described by Ahmed et al. [12], which mediated effective elimination of B‐LCLs in vivo.

EBV is known to employ different strategies to avoid immune recognition and persist in the host. For example, EBV‐infected (malignant) cells can release a viral IL‐10 homologue to suppress a pro‐inflammatory cytokine environment [16]. For HL, IL‐12 released by EBV‐specific T cells could be shown to counteract the TME, likely by antagonising downregulation of T‐bet and the Th1 transcriptional programme [17]. Moreover, next to T cells, the importance of NK cells was demonstrated for the control of different EBV‐driven pathologies since primary immunodeficiencies in NK cells predispose patients to EBV‐driven pathologies [18] and a distinct subpopulation of tonsillar NK cells could restrict B‐cell transformation by EBV [19]. Since iIL‐12‐ and iIL‐18_TRUCKs were shown to potently mediate recruitment and activation of NK cells [21, 22], LMP2A_TRUCKs were generated by combination of the constitutive LMP2A_CAR expression with inducible IL‐12 or IL‐18 expression in an ‘All‐in‐One vector’ as described for GD2‐specific iIL‐12 and iIL‐18 TRUCKs [24] to improve their efficacy and mediate bystander immune cell recruitment in the TME of EBV‐associated malignancies. Functionality of the inducible cassette could be shown by significant upregulation of EGFP, IL‐12 or IL‐18 by corresponding LMP2A_TRUCKs following recognition of A02_CLG^+^ target cells. In that, IL‐12 release was induced in higher amounts than IL‐18 by corresponding TRUCKs, which is in accordance with previous studies using the same inducible cassette design [24, 31, 37].

In co‐cultures with A02_CLG^+^ target cells, LMP2A_iIL‐12_TRUCKs exhibited potent T‐cell activation, the release of effector molecules, and cytotoxic activity. In fact, the release of soluble mediators such as IFN‐γ, IL‐4, IL‐6, TNF‐α and granulysin, as well as the reduction of target cell viability following co‐culture with T2_CLG or SPI_A02_CLG, was even augmented for LMP2A_iIL‐12_TRUCKs when compared with LMP2A_iIL‐18_TRUCKs. This is in accordance with other studies in which pre‐conditioning of naïve CD8^+^ T cells with IL‐12 in vitro led to potent anti‐tumour performance in tumour‐bearing mice [38, 39], and studies involving TRUCKs with inducible IL‐12 reporting increased TNF‐α and IFN‐γ release when compared with corresponding CAR‐Ts of the second generation [13, 21].

The T2 and SPI target cell models used in this study allowed us to decipher HLA and peptide specificity of the generated LMP2A‐targeting T cells in detail and to detect off‐target reactions against control peptides or naturally occurring HLA‐A*02:01 (or HLA‐A*01:01) epitopes. Unexpectedly, the models revealed off‐target effects for LMP2A_iIL‐12_TRUCKs as they mediated unspecific elimination of SPI_A01, SPI_A02, and MCF‐7 cells independent of the presence of CLG peptide, while all other LMP2A‐specific T cells were highly specific for A02_CLG. Interestingly, Maus et al. developed TCR‐like CAR‐Ts based on a TCR‐like antibody targeting a peptide derived from the cancer‐testis antigen NY‐ESO‐1 presented in the context of HLA‐A*02:01 and observed off‐target elimination of HLA‐A*02:01 ^*+*^, but NY‐ESO‐1 peptide‐negative cells and attributed it to an increased avidity of the CAR compared to the TCR‐like antibody [40]. However, loss of target specificity was not observed for an affinity‐matured NY‐ESO‐1 TCR‐like CAR in a different study [15]. Also, in the current study, LMP2A_CAR‐Ts and LMP2A_iIL‐18_TRUCKs did not show any cross‐reactivity, neither against the control peptide SLL nor the HLA‐A*02:01 itself. Also, reactivity of LMP2A_CAR_iIL‐12 transduced reporter cells was strictly A02_CLG specific. Thus, it is unlikely that the off‐target response by LMP2A_iIL‐12_TRUCKs was caused by the affinity of the scFv, but rather due to the phenotypical changes mediated by IL‐12 during generation. Activation via anti‐CD3/CD28 and supplementation with IL‐7 and IL‐15 is also part of clinical manufacturing protocols for approved CD19‐targeting CAR‐Ts [41], but led to increased IL‐12 concentrations in the culture supernatant of LMP2A_iIL‐12_TRUCKs. As for IL‐12 pre‐conditioned Delta_CAR‐Ts and LMP2A_CAR‐Ts, this drove them towards a predominantly T_EM_ memory phenotype with a significant proportion of NK‐like CD56^+^CD94^+^CD62L^+^ cells and an increased release of IFN‐γ and IL‐6, as well as expression of LAG‐3, TIM‐3 and CD39.

Others investigated that IL‐12 alone or synergistically in combination with IL‐18 can lower the threshold required for activation of CD8^+^ T cells [42]. Since this effect was dependent on IL‐12, IL‐18 alone did not force a threshold shift. These findings suggest that the IL‐12 released by the LMP2A_iIL‐12_TRUCKs induces a phenotype in which they react already to marginal stimuli, and which resulted in cytotoxicity towards target cells only by recognising transduced HLA molecules on T2 and SPI‐801 cells, as well as HLA‐A*02 on MCF‐7 cells. Interestingly, LMP2A_iIL‐12_TRUCKs, although generated from CD8^+^ T cells of HLA‐A*02:01 ^*+*^ individuals, did not show any sign of CAR‐mediated fratricide that was more than reduced viabilities of IL‐12 pre‐conditioned Delta_CAR‐Ts or LMP2A_CAR‐Ts. One potential explanation would be the phenotypical reprogramming of LMP2A_iIL‐12_TRUCKs, including the NK‐like signature by IL‐12 signalling. This was observed for CAR‐Ts with an integrated IL‐12 exodomain and enabled elimination of antigen‐negative cancer cells [33]. However, as LMP2A_iIL‐12_TRUCKs (or IL‐12 pre‐conditioned cells) did not react towards HLA‐negative Daudi and SPI cells, it is more likely not an NK‐like response, but a TCR‐mediated alloreactive response that was boosted by the described phenotypical changes. In future experiments, only detailed evaluation of this A02_CLG independent reactivity of LMP2A_iIL‐12_TRUCKs, as well as fine‐tuning of the inducible promoter to reduce IL‐12 leakage and phenotypical changes during generation could make these cells suitable for clinical application to treat EBV‐associated malignancies. Regarding the clinical development of all LMP2A_CAR‐Ts and LMP2A_TRUCKs, the co‐expressed EGFRt, used as a selection and enrichment marker in the present study, could serve as a potential suicide marker and enable CAR‐T depletion following administration of cetuximab for increased safety [43].

In contrast, LMP2A_CAR‐Ts and LMP2A_iIL‐18_TRUCKs were highly target‐specific, with effector responses restricted to A02_CLG^+^ cells. In that, LMP2A_iIl‐18_TRUCKs exhibited slightly higher IFN‐γ release accompanying the IL‐18 secretion during expansion, as well as an increased target‐induced CD25 expression, slightly higher release of IL‐2, IL‐4, sFasL, TNF‐α, perforin and granulysin, and a more immediate killing capacity than LMP2A_CAR‐Ts following co‐culture with T2_CLG or SPI_A02_CLG cells. This is in line with previous studies reporting superior antitumor activity of CAR‐ and TCR‐engineered T cells with iIL‐18 release in vivo [22, 37, 44, 45, 46]. In detail, the release of IL‐18 was shown to convert (engineered) T cells into effectors with an inflammatory T‐bet^high^ FoxO1^low^ phenotype [22], an augmented IFN‐γ release and higher proliferation capacity [44] as well as an improved killing capacity in vitro [31]. Additional to these effects, LMP2A_iIL‐18_TRUCKs are expected to exhibit superior anti‐tumour efficacy than LMP2A_CAR‐Ts in vivo due to TME re‐modulation, immune evasion counteracting, and attraction of additional immune cells. The potential of iIL‐18_TRUCKs to modulate the TME of solid tumours have been demonstrated by induced changes of the bystander immune cell composition towards higher numbers of M1 macrophages and NKG2D^+^ NK cells, as well as reduced numbers of regulatory T cells, suppressive dendritic cells (DCs) and M2 macrophages [22]. Also for IL‐18‐secreting CD19‐CAR‐Ts, an increased expansion of immune effector cells, including NK cells, NKT cells, DCs, and endogenous CD8^+^ T cells, was observed at the tumour site [45].

To conclude, we here developed LMP2A_CAR‐Ts and LMP2A_iIL‐18_TRUCKs that exhibit efficient and target‐specific killing capacity towards A02_CLG^+^ target cells. Due to the restricted availability of the A02_CLG target only on EBV‐infected cells, off‐target side effects impeding the general immunity, in contrast to classical treatment options that also affect the healthy B cell compartment, are not expected. LMP2A_iIL‐18_TRUCKs, especially in situations in which treatment with EBV‐specific memory T cells [32, 34, 35, 36] or CD19‐CAR‐Ts [8] fails due to the immunosuppressive TME, promise benefit to overcome EBV‐mediated immune evasion strategies, re‐modulate the anti‐inflammatory cytokine environment and moreover attract NK cells that are generally rare in proximity to tumour cells. This makes them especially promising for EBV‐associated malignancies in already immunocompromised patients, including EBV‐associated PTLD in transplant patients, HL and other lymphomas in patients with HIV, as well as non‐HL in patients with primary immune disorders. Thus, LMP2A_CAR‐Ts and especially LMP2A_iIL‐18_TRUCKs may serve as a promising new tool to treat various EBV‐associated malignancies.

## Author Contributions

**Anna Christina Dragon:** conceptualisation, methodology, software, validation, formal analysis, investigation, data curation, writing – original draft preparation, writing – review and editing, visualisation, supervision, project administration. **Stefanie Thoelke:** methodology, software, validation, formal analysis, investigation, data curation, writing – original draft preparation, writing – review and editing, visualisation. **Philip Mausberg:** methodology, writing – review and editing. **Katharina Zimmermann:** methodology, writing – review and editing. **Rainer Blasczyk:** resources, writing – review and editing. **Michael Hudecek:** resources, writing – review and editing. **Hinrich Abken:** methodology, writing – review and editing. **Axel Schambach:** methodology, resources, writing – review and editing. **Britta Maecker‐Kolhoff:** writing – review and editing, supervision. **Britta Eiz‐Vesper:** conceptualisation, validation, formal analysis, investigation, resources, data curation, writing – review and editing, supervision, project administration, funding acquisition.

## Ethics Statement

All experiments involving primary human cells were performed using residual blood samples from routine platelet collection (Institute of Transfusion Medicine and Transplant Engineering, Hannover Medical School, Germany). Written informed consent was obtained from all donors as approved by the Ethics Committee of Hannover Medical School (2519–2014, 3639–2017).

## Conflicts of Interest

The authors A.S., H.A. and K.Z. have an active patent for ‘All‐in‐One vector for car and therapeutic effector molecule’ (EP3986428A1). M.H. is listed as an inventor on patent applications and granted patents related to CAR‐T technologies that have been filed by the Fred Hutchinson Cancer Research Center, Seattle, WA (M.H.) and the University of Würzburg, Würzburg, Germany (M.H.) and that have been, in part, licenced by industry. M.H. is a co‐founder and equity owner of T‐CURX GmbH, Würzburg, Germany. M.H. received speaker honoraria from BMS, Janssen, and Kite/Gilead. Apart from that, the authors declare that the research was conducted in the absence of any commercial or financial relationships that could be construed as a potential conflicts of interest.

## Supporting information


**Figure S1:** Frequency of transduced JE6‐1 reporter cells and HLA staining of target cells. (A–D) All constructs were transduced into a JE6‐1 derived reporter cell line. The frequency of transduced cells and transgene expression levels were determined by staining with (A, B) biotin‐anti‐EGFRt and PE‐streptavidin or (C, D) biotin‐anti‐G_4_S and PE‐streptavidin, whereby (A, C) the frequency of positive cells as well as (B, D) the mean fluorescence intensity (MFI) were analysed. Data are shown as mean + SD, whereby each point represents an independent experiment ((A, B) *n* = 6–12, (C, D) *n* = 3). (E) As target cells for the evaluation of LMP2A_CAR constructs, SPI‐801 cells were transduced to express HLA‐A*02:01, and transduced cells enriched (SPI_A02). Expression of HLA‐A*02 was confirmed by staining with anti‐HLA‐A*02 antibody before and after enrichment. (F) Further target cells were stained for HLA‐A*02 and HLA‐ABC. (E, F) Data are shown as representative dot plots.
**Figure S2:** The LMP2A‐targeting constructs were transduced into a JE6‐1‐derived reporter cell line. A Delta_CAR construct lacking the LMP2A‐specific scFv, as well as a CD19_CAR construct with a CD19‐targeting scFv served as controls. Transduced reporter cells were co‐cultured with the indicated target cells, whereby A02‐ indicates cells derived from HLA‐A*02:01 ^*−*^ and A02+ from HLA‐A*02:01 ^*+*^ individuals, _CLG indicates cells loaded with the LMP2A‐derived peptide CLGGLLTMV, _SLL indicates cells loaded with the PRAME‐derived control peptide SLLQHLIGL, _A02 indicates cells transduced with HLA‐A*02:01 and _A01 cells transduced with HLA‐A*01:01. (E, G) To stabilise HLA expression on T2 cells, soluble β_2_ microglobulin (b2m) was added to these cells 16–24 h before co‐cultures. T2 cells without b2m addition (w/o b2m) served as controls. (J) Anti‐CD3/CD28 stimulation of (transduced) reporter cells served as positive control. (A–J) After 24 h in an E:T ratio of 1:1, specific upregulation of (A, C–G, I, J) EGFP indicating NFAT activity and (B, H) ECFP indicating NF‐κB activity was calculated by subtracting the EGFP or ECFP expression of transduced reporter cells cultured alone from the respective expression of reporter cells co‐cultured with target cells. Data are shown as mean + SD, whereby each point represents an independent experiment ((A) *n* = 5–9, (B, C) *n* = 4–5, (D) *n* = 3–5, (E) *n* = 5, (F) *n* = 3, (G, H) *n* = 2–3, (I) *n* = 3, (J) *n* = 3–5). Statistical analysis was performed using two‐way ANOVA and (A, D–J) Tukey's or (B, C) Šídák's multiple comparisons test. Only significant differences to untransduced reporter cells co‐cultured with the same target cells are shown. *p ≤ 0.05, **p ≤ 0.01, ***p ≤ 0.001, ****p ≤ 0.0001. (A, C–G, I, J) LMP2A_CAR_iEGFP constructs were not evaluated for NFAT activation capacity due to the interference of the EGFP reporter signal with the inducible EGFP cassette.
**Figure S3:** Analysis of LMP2A_CAR‐Ts and LMP2A_TRUCKs during expansion. The LMP2A‐targeting constructs were transduced into CD8^+^ T cells isolated from healthy individuals. Respective untransduced T cells (untransduced Ts) served as control. (A) T‐cell expansion was determined by cell counting and is shown as fold increase relative to the respective cell numbers on Day 0 (left) or on Days 8–9 after enrichment (right) of transduced cells. Data are shown as mean + SD (*n* = 8). (B) After further expansion following enrichment of EGFRt^+^ cells, CAR expression on LMP2A_CAR‐Ts and LMP2A‐TRUCKs was determined by staining with biotin‐anti‐G_4_S and PE‐streptavidin. Data are shown as representative dot plots of flow cytometric analysis. (C) Phenotype of LMP2A_CAR‐Ts and TRUCKs during expansion was assessed on the indicated days as naïve (T_N_: CD45RO^−^ CCR7^+^ CD95^−^), stem‐cell memory (T_SCM_: CD45RO^−^ CCR7^+^ CD95^+^), central memory (T_CM_: CD45RO^+^ CCR7^+^), effector memory (T_EM_: CD45RO^+^ CCR7^−^), and effector (T_EF_: CD45RO^−^ CCR7^−^) T cells. Data are shown as mean (*n* = 2–4). (D–K) The indicated markers for exhaustion and activation were analysed during expansion using flow cytometry. Data are shown as mean + SD (*n* = 5–8). Statistical analysis was performed using two‐way ANOVA and Tukey's multiple comparisons test. Significant differences to values obtained for untransduced Ts at the same day are indicated. **p ≤ 0.01, ****p ≤ 0.0001. d = Day; MFI = mean fluorescence intensity.
**Figure S4:** LMP2A_CAR‐Ts and LMP2A_TRUCKs are specifically activated following recognition of A02_CLG^+^ T2 cells. LMP2A_CAR‐Ts and LMP2A_TRUCKs were either cultured alone (w/o target) or co‐cultured with T2 cells, T2 cells loaded with the LMP2A‐derived peptide CLGGLLTMV (T2_CLG), T2 cells loaded with the PRAME‐derived control peptide SLLQHLIGL (T2_SLL) or CLG‐loaded T2 cells with HLA‐A*02:01 knockout (T2^A02ko^_CLG), T2 cells transduced with HLA‐A*01:01 (T2_A01) or CLG‐loaded T2_A01 with HLA‐A*02:01 knockout (T2_A01^A02ko^_CLG) for 48 h in an E:T ratio of 1:1. Corresponding co‐cultures with Delta_CAR‐Ts lacking the LMP2A‐specific scFv or CD19_CAR‐Ts with a CD19‐targeting scFv served as controls. Markers for T‐cell activation were determined by flow cytometry and are shown as (A, C) frequency of positive cells or (B) mean fluorescence intensity (MFI). Data are shown as mean + SD, whereby each point represents individual experiment (*n* = 3–5 with T cells from *n* = 3–4 donors). Statistical analysis was performed using two‐way ANOVA and Tukey's multiple comparisons test. *p ≤ 0.05, **p ≤ 0.01, ***p ≤ 0.001, ****p ≤ 0.0001.
**Figure S5:** LMP2A_CAR‐Ts and LMP2A_iIL‐18_TRUCKs are specifically activated following recognition of A02_CLG^+^ SPI cells, whereas LMP2A_iIL‐12_TRUCKs also respond to A02^+^ or A01^+^ target cells. LMP2A_CAR‐Ts and LMP2A_TRUCKs were either cultured alone (w/o target) or co‐cultured with untransduced SPI‐801 cells (SPI), SPI transduced with HLA‐A*02:01 either unloaded (SPI_A02), SLL‐loaded (SPI_A02_SLL), CLG‐loaded (SPI_A02_CLG), SPI transduced with HLA‐A*01:01 (SPI_A01) or CLG‐loaded SPI_A01 (SPI_A01_CLG) for 48 h in an E:T ratio of (A, B, D‐F) 1:1 or (C) 0.5:1. (E, F) Corresponding co‐cultures with Delta_CAR‐Ts lacking the LMP2A‐specific scFv or CD19_CAR‐Ts with a CD19‐targeting scFv served as controls. (A–F) Markers for T‐cell activation were determined by flow cytometry and are shown as (A, C–E) mean fluorescence intensity (MFI) or (B, F) frequency of positive cells. Data are shown as mean + SD, whereby each point represents individual experiment ((A–D) *n* = 4–6 donors, (E, F) *n* = 3–5 with T cells from *n* = 3–4 donors). Statistical analysis was performed using two‐way ANOVA and Tukey's multiple comparisons test. *p ≤ 0.05, **p ≤ 0.01, ***p ≤ 0.001, ****p ≤ 0.0001.
**Figure S6:** LMP2A_CAR‐Ts and LMP2A_iIL‐18_TRUCKs release pro‐inflammatory mediators following recognition of A02_CLG^+^ target cells, whereas LMP2A_iIL‐12_TRUCKs also respond to A02^+^ target cells. LMP2A_CAR‐Ts and LMP2A_TRUCKs were either cultured alone (w/o target) or co‐cultured with (A) T2 cells transduced with HLA‐A*01:01 (T2_A01), or CLG‐loaded T2_A01 with HLA‐A*02:01 knockout (T2_A01^A02ko^_CLG) or (B–D) untransduced SPI‐801 cells (SPI), SPI transduced with HLA‐A*02:01 either unloaded (SPI_A02), CLG‐loaded (SPI_A02_CLG) or SLL‐loaded (SPI_A02_SLL) for 48 h in an E:T ratio of (A, B, D) 1:1 or (C) 0.5:1. (A) Corresponding co‐cultures with Delta_CAR‐Ts lacking the LMP2A‐specific scFv or CD19_CAR‐Ts with a CD19‐targeting scFv served as controls. (A, B) The concentration of different mediators in the culture supernatants was analysed by a bead‐based multiplex cytokine profiling using flow cytometry. Data are shown as mean ((A) *n* = 3–5 with T cells from *n* = 3–4 donors, (B) *n* = 6–8 donors). Statistical analysis was performed using two‐way ANOVA and Tukey's multiple comparisons test. Significant differences to values obtained for untransduced Ts co‐cultured with the same target cells are indicated. 12 = LMP2A_iIL‐12_TRUCKs, 18 = LMP2A_iIL‐18_TRUCKs, 19 = CD19_CAR‐Ts, C = LMP2A_CAR‐Ts, D = Delta_CAR‐Ts, E = LMP2A_iEGFP_TRUCKs, *ø* = untransduced Ts. (C) The cytotoxic activity of LMP2A_CAR‐Ts and LMP2A_TRUCKs was determined by analysing the viability of CTV‐labelled target cells by 7‐AAD staining and subsequent flow cytometry analysis. Data are shown as representative plots for the gating of viable target cells. (D). Cell death in co‐cultures was confirmed by analysis of LDH release into the co‐culture supernatant. LDH levels are expressed in % of the maximum lysis level obtained using controls lysed with 1% Triton X‐100. Data are shown as mean + SD, whereby each point represents one donor (*n* = 6). Statistical analysis was performed using two‐way ANOVA and Tukey's multiple comparisons test. Only significant differences to untransduced T cells co‐cultured with the same target cells are shown. *p ≤ 0.05, **p ≤ 0.01, ***p ≤ 0.001, ****p ≤ 0.0001.
**Figure S7:** IL‐12 pre‐conditioning of T cells drives them towards T_EM_ and an NK‐like phenotype that is similar to LMP2A_iIL‐12_TRUCKs. All constructs were transduced into CD8^+^ T cells isolated from healthy individuals. Respective untransduced T cells served as control. To evaluate impact of IL‐12 or IL‐18 pre‐conditioning, these cytokines were exogenously added to untransduced T cells (IL‐12_ or IL‐18_untransduced Ts), Delta_CAR‐Ts (IL‐12_ or IL‐18_Delta_CAR‐Ts) and LMP2A_CAR‐Ts (IL‐12_ or IL‐18_LMP2A_CAR‐Ts) during generation. (A, E) T‐cell memory phenotype was assessed on Day 15 as naïve (T_N_: CD45RO^−^ CCR7^+^ CD95^−^), stem‐cell memory (T_SCM_: CD45RO^−^ CCR7^+^ CD95^+^), central memory (T_CM_: CD45RO^+^ CCR7^+^), effector memory (T_EM_: CD45RO^+^ CCR7^−^) and effector (T_EF_: CD45RO^−^ CCR7^−^) T cells. Data are shown as mean. (B, F) T‐cell expansion was determined by cell counting and is shown as fold increase relative to the respective cell numbers on Day 0 (left) or on Day 9 after enrichment of transduced cells (right). (B, G) Viability of T cells as assessed by 7‐AAD staining, as well as (D, H) frequency of cells with an NK‐like phenotype (CD56^+^CD94^+^CD62L^+^) was assessed on the indicated days using flow cytometry. (B–D, F–H) Data are shown as mean + SD (*n* = 3–4). Statistical analysis was performed using two‐way ANOVA and Tukey's multiple comparisons test. Significant differences to values obtained for (B, F) untransduced Ts or (C, D, G, H) corresponding cells not treated with IL‐12 or IL‐18 at the same day are indicated. *p ≤ 0.05, **p ≤ 0.01, ****p ≤ 0.0001. D = day.
**Figure S8:** IL‐12 pre‐conditioning of transduced T cells induces reactivity towards SPI_A01, SPI_A02 and MCF‐7 cells that is similar to LMP2A_iIL‐12_TRUCKs. To evaluate impact of IL‐12 or IL‐18 pre‐conditioning, these cytokines were exogenously added to untransduced T cells (IL‐12_ or IL‐18_untransduced Ts), Delta_CAR‐Ts (IL‐12_ or IL‐18_Delta_CAR‐Ts) and LMP2A_CAR‐Ts (IL‐12_ or IL‐18_LMP2A_CAR‐Ts) during generation from CD8^+^ T cells isolated from healthy individuals. Their target response was compared to LMP2A_iIL‐12_TRUCKs. All T cells were either cultured alone (w/o target) or co‐cultured with SPI transduced with HLA‐A*01:01 (SPI_A01) or HLA‐A*02:01 (SPI_A02), Daudi or MCF‐7 cells for 48 h in an E:T ratio of 1:1. (A, B) Markers for T‐cell activation were determined by flow cytometry and are shown as (A) mean fluorescence intensity (MFI) or (B) frequency of positive cells. (C) The concentration of different mediators in the culture supernatants was analysed by a bead‐based multiplex cytokine profiling using flow cytometry. Significant differences to values obtained for untransduced Ts co‐cultured with the same target cells are indicated. For untransduced Ts (*ø*), Delta_CAR‐Ts (D) and LMP2A_CAR‐Ts (C), conditions without cytokine supplementation (−), with IL‐12 pre‐conditioning (12) or IL‐18 pre‐conditioning (18) were compared. LMP2A_iIL‐12_TRUCKs (12*) were not pre‐conditioned. (D, E) The cytotoxic activity of transduced T cells was determined by analysing the viability of CTV‐labelled target cells by 7‐AAD staining and subsequent flow cytometry analysis. Target cell viability was normalised to viabilities of corresponding target cells cultured alone. Data are shown as (A, B, D) mean + SD or (C) mean, whereby each point represents one individual experiment (*n* = 3–5 with T cells from *n* = 3–4 donors). Statistical analysis was performed using two‐way ANOVA and Tukey's multiple comparisons test. Only significant differences to untransduced T cells and corresponding cells not treated with IL‐12 or IL‐18 when co‐cultured with the same target cells are indicated. (A, B, D) For SPI‐A01 cells, data from co‐cultures with untransduced T cells, Delta_CAR‐Ts, LMP2A_CAR‐Ts and LMP2A_iIL‐12‐TRUCKs are the same as in Figures S5E,F and 5E, as those were obtained in the same experiments and are repeated here for comparison with the pre‐conditioned counterparts. *p ≤ 0.05, **p ≤ 0.01, ***p ≤ 0.001, ****p ≤ 0.0001.
**Table S1:** Utilised cell lines and cultivation. RPMI 1640 medium (Lonza), foetal bovine serum (FBS; Merck), L‐glutamine (c.c.pro), DMEM (Lonza).
**Table S2:** Antibodies used for flow cytometry. Peridinin‐chlorophyll‐protein (PerCP), Alexa Fluor (AF), allophycocyanin (APC), Brilliant Violet (BV), phycoerythrin (PE), fluorescein isothiocyanate (FITC).

## Data Availability

The data that support the findings of this study are available from the corresponding author upon reasonable request.
