# Supplementary material for: LMP2A‐Targeting CAR‐T Cells Equipped With Inducible IL‐18 to Address EBV‐Associated Malignancies
Source: HLA. 2025 Oct 21;106(4):e70439. doi: 10.1111/tan.70439 (PMC12538270; doi:10.1111/tan.70439)
Supplement: Supplementary file 1 — Figure S1: Frequency of transduced JE6‐1 reporter cells and HLA staining of target cells. (A–D) All constructs were transduced into a JE6‐1 derived reporter cell line. The frequency of transduced cells and transgene expression levels were determined by staining with (A, B) biotin‐anti‐EGFRt and PE‐streptavidin or (C, D) biotin‐anti‐G4S and PE‐streptavidin, whereby (A, C) the frequency of positive cells as well as (B, D) the mean fluorescence intensity (MFI) were analysed. Data are shown as mean + SD, whereby each point represents an independent experiment ((A, B) n = 6–12, (C, D) n = 3). (E) As target cells for the evaluation of LMP2A_CAR constructs, SPI‐801 cells were transduced to express HLA‐A*02:01, and transduced cells enriched (SPI_A02). Expression of HLA‐A*02 was confirmed by staining with anti‐HLA‐A*02 antibody before and after enrichment. (F) Further target cells were stained for HLA‐A*02 and HLA‐ABC. (E, F) Data are shown as representative dot plots. Figure S2: The LMP2A‐targeting constructs were transduced into a JE6‐1‐derived reporter cell line. A Delta_CAR construct lacking the LMP2A‐specific scFv, as well as a CD19_CAR construct with a CD19‐targeting scFv served as controls. Transduced reporter cells were co‐cultured with the indicated target cells, whereby A02‐ indicates cells derived from HLA‐A*02:01 − and A02+ from HLA‐A*02:01 + individuals, _CLG indicates cells loaded with the LMP2A‐derived peptide CLGGLLTMV, _SLL indicates cells loaded with the PRAME‐derived control peptide SLLQHLIGL, _A02 indicates cells transduced with HLA‐A*02:01 and _A01 cells transduced with HLA‐A*01:01. (E, G) To stabilise HLA expression on T2 cells, soluble β2 microglobulin (b2m) was added to these cells 16–24 h before co‐cultures. T2 cells without b2m addition (w/o b2m) served as controls. (J) Anti‐CD3/CD28 stimulation of (transduced) reporter cells served as positive control. (A–J) After 24 h in an E:T ratio of 1:1, specific upregulation of (A, C–G, I, J) EGFP indicating [file TAN-106-e70439-s001.docx]

APPENDIX to: LMP2A-Targeting CAR-T Cells Equipped With Inducible IL-18 to Address EBV-Associated Malignancies

**Supplementary Figures**


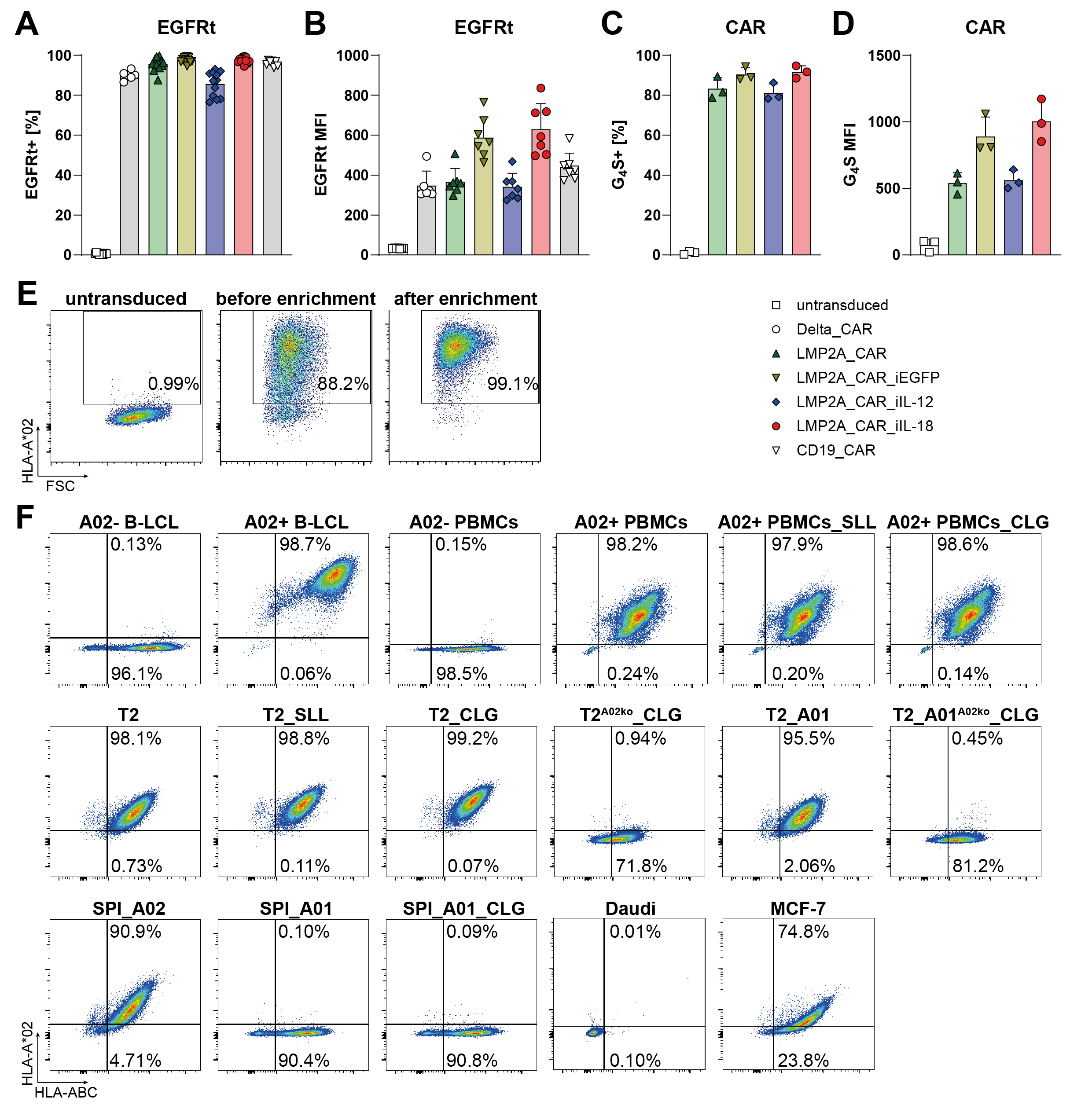


**Figure S1:** Frequency of transduced JE6‐1 reporter cells and HLA staining of target cells. (A–D) All constructs were transduced into a JE6‐1 derived reporter cell line. The frequency of transduced cells and transgene expression levels were determined by staining with (A, B) biotin‐anti‐EGFRt and PE‐streptavidin or (C, D) biotin‐anti‐G_4_S and PE‐streptavidin, whereby (A, C) the frequency of positive cells as well as (B, D) the mean fluorescence intensity (MFI) were analysed. Data are shown as mean + SD, whereby each point represents an independent experiment ((A, B) *n* = 6–12, (C, D) *n* = 3). (E) As target cells for the evaluation of LMP2A_CAR constructs, SPI‐801 cells were transduced to express *HLA‐A*02:01*, and transduced cells enriched (SPI_A02). Expression of *HLA‐A*02* was confirmed by staining with anti‐*HLA‐A*02* antibody before and after enrichment. (F) Further target cells were stained for *HLA‐A*02* and HLA‐ABC. (E, F) Data are shown as representative dot plots.


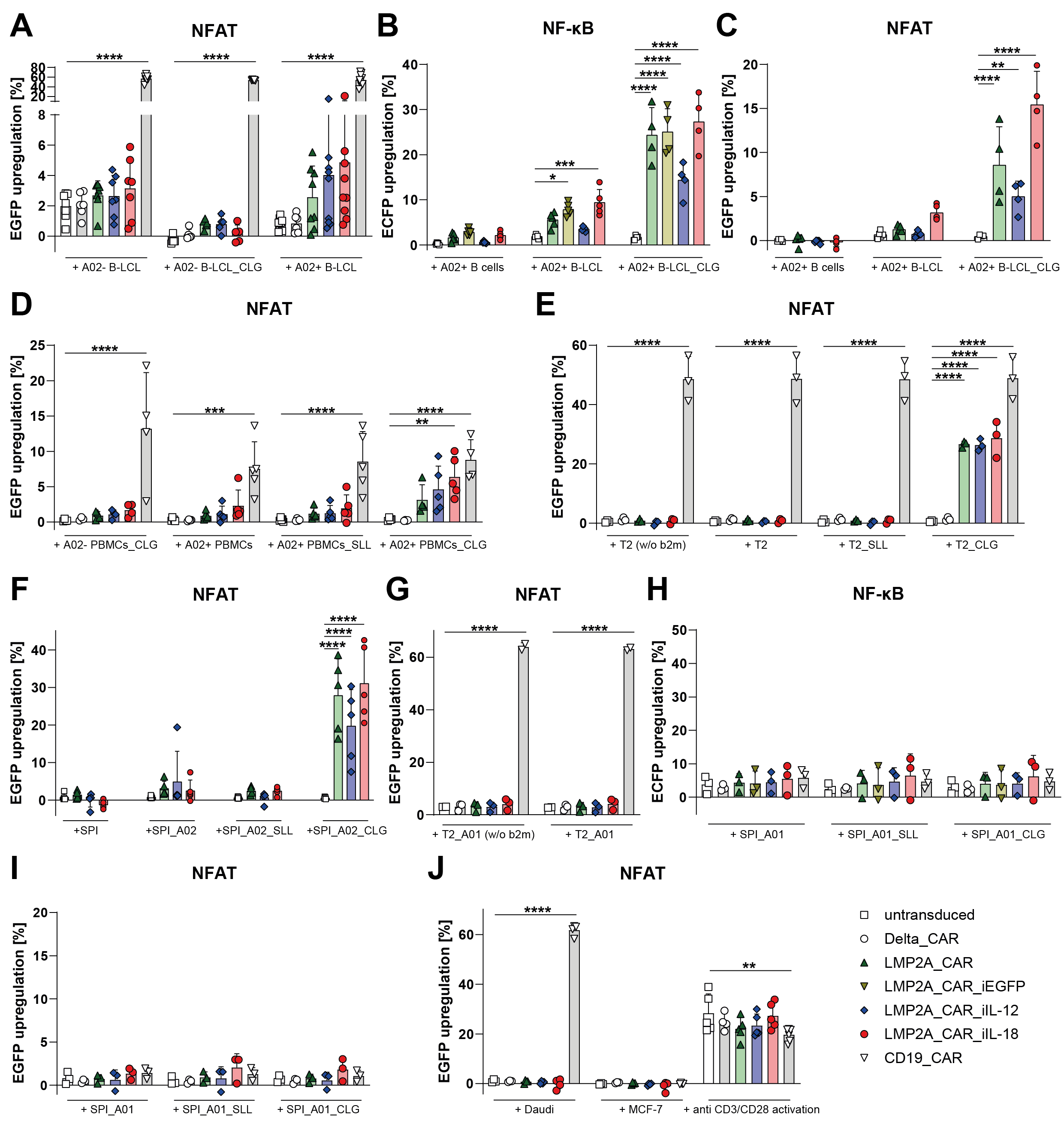


**Figure S2:** The LMP2A‐targeting constructs were transduced into a JE6‐1‐derived reporter cell line. A Delta_CAR construct lacking the LMP2A‐specific scFv, as well as a CD19_CAR construct with a CD19‐targeting scFv served as controls. Transduced reporter cells were co‐cultured with the indicated target cells, whereby A02‐ indicates cells derived from *HLA‐A*02:01^−^* and A02+ from *HLA‐A*02:01^+^* individuals, _CLG indicates cells loaded with the LMP2A‐derived peptide CLGGLLTMV, _SLL indicates cells loaded with the PRAME‐derived control peptide SLLQHLIGL, _A02 indicates cells transduced with *HLA‐A*02:01* and _A01 cells transduced with *HLA‐A*01:01*. (E, G) To stabilise HLA expression on T2 cells, soluble β_2_ microglobulin (b2m) was added to these cells 16–24 h before co‐cultures. T2 cells without b2m addition (w/o b2m) served as controls. (J) Anti‐CD3/CD28 stimulation of (transduced) reporter cells served as positive control. (A–J) After 24 h in an E:T ratio of 1:1, specific upregulation of (A, C–G, I, J) EGFP indicating NFAT activity and (B, H) ECFP indicating NF‐κB activity was calculated by subtracting the EGFP or ECFP expression of transduced reporter cells cultured alone from the respective expression of reporter cells co‐cultured with target cells. Data are shown as mean + SD, whereby each point represents an independent experiment ((A) *n* = 5–9, (B, C) *n* = 4–5, (D) *n* = 3–5, (E) *n* = 5, (F) *n* = 3, (G, H) *n* = 2–3, (I) *n* = 3, (J) *n* = 3–5). Statistical analysis was performed using two‐way ANOVA and (A, D–J) Tukey's or (B, C) Šídák's multiple comparisons test. Only significant differences to untransduced reporter cells co‐cultured with the same target cells are shown. **p* ≤ 0.05, ***p* ≤ 0.01, ****p* ≤ 0.001, *****p* ≤ 0.0001. (A, C–G, I, J) LMP2A_CAR_iEGFP constructs were not evaluated for NFAT activation capacity due to the interference of the EGFP reporter signal with the inducible EGFP cassette.

**
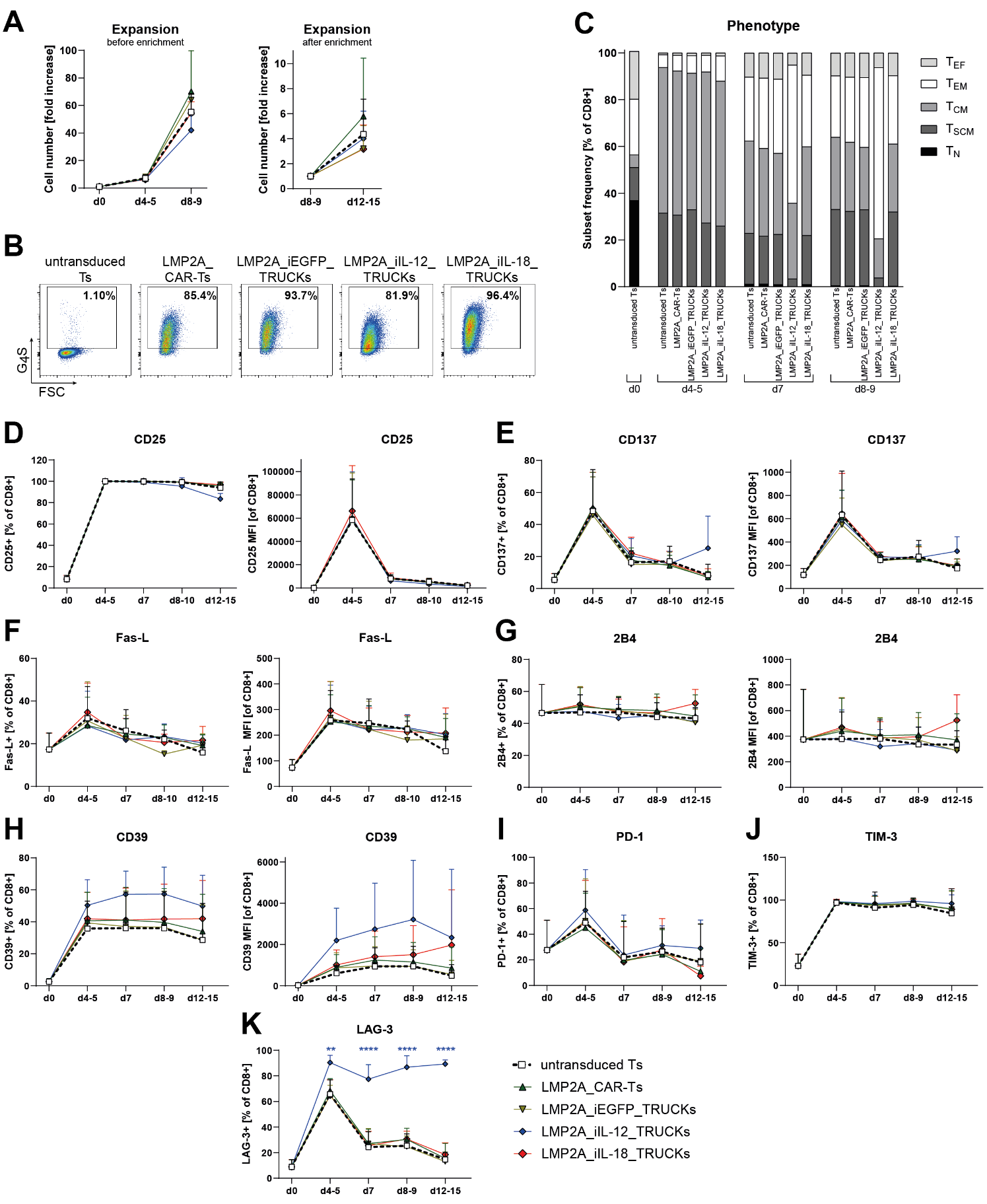
**

**Figure S3:** Analysis of LMP2A_CAR‐Ts and LMP2A_TRUCKs during expansion. The LMP2A‐targeting constructs were transduced into CD8^+^ T cells isolated from healthy individuals. Respective untransduced T cells (untransduced Ts) served as control. (A) T‐cell expansion was determined by cell counting and is shown as fold increase relative to the respective cell numbers on Day 0 (left) or on Days 8–9 after enrichment (right) of transduced cells. Data are shown as mean + SD (*n* = 8). (B) After further expansion following enrichment of EGFRt^+^ cells, CAR expression on LMP2A_CAR‐Ts and LMP2A‐TRUCKs was determined by staining with biotin‐anti‐G_4_S and PE‐streptavidin. Data are shown as representative dot plots of flow cytometric analysis. (C) Phenotype of LMP2A_CAR‐Ts and TRUCKs during expansion was assessed on the indicated days as naïve (T_N_: CD45RO^−^ CCR7^+^ CD95^−^), stem‐cell memory (T_SCM_: CD45RO^−^ CCR7^+^ CD95^+^), central memory (T_CM_: CD45RO^+^ CCR7^+^), effector memory (T_EM_: CD45RO^+^ CCR7^−^), and effector (T_EF_: CD45RO^−^ CCR7^−^) T cells. Data are shown as mean (*n* = 2–4). (D–K) The indicated markers for exhaustion and activation were analysed during expansion using flow cytometry. Data are shown as mean + SD (*n* = 5–8). Statistical analysis was performed using two‐way ANOVA and Tukey's multiple comparisons test. Significant differences to values obtained for untransduced Ts at the same day are indicated. ***p* ≤ 0.01, *****p* ≤ 0.0001. d = Day; MFI = mean fluorescence intensity.


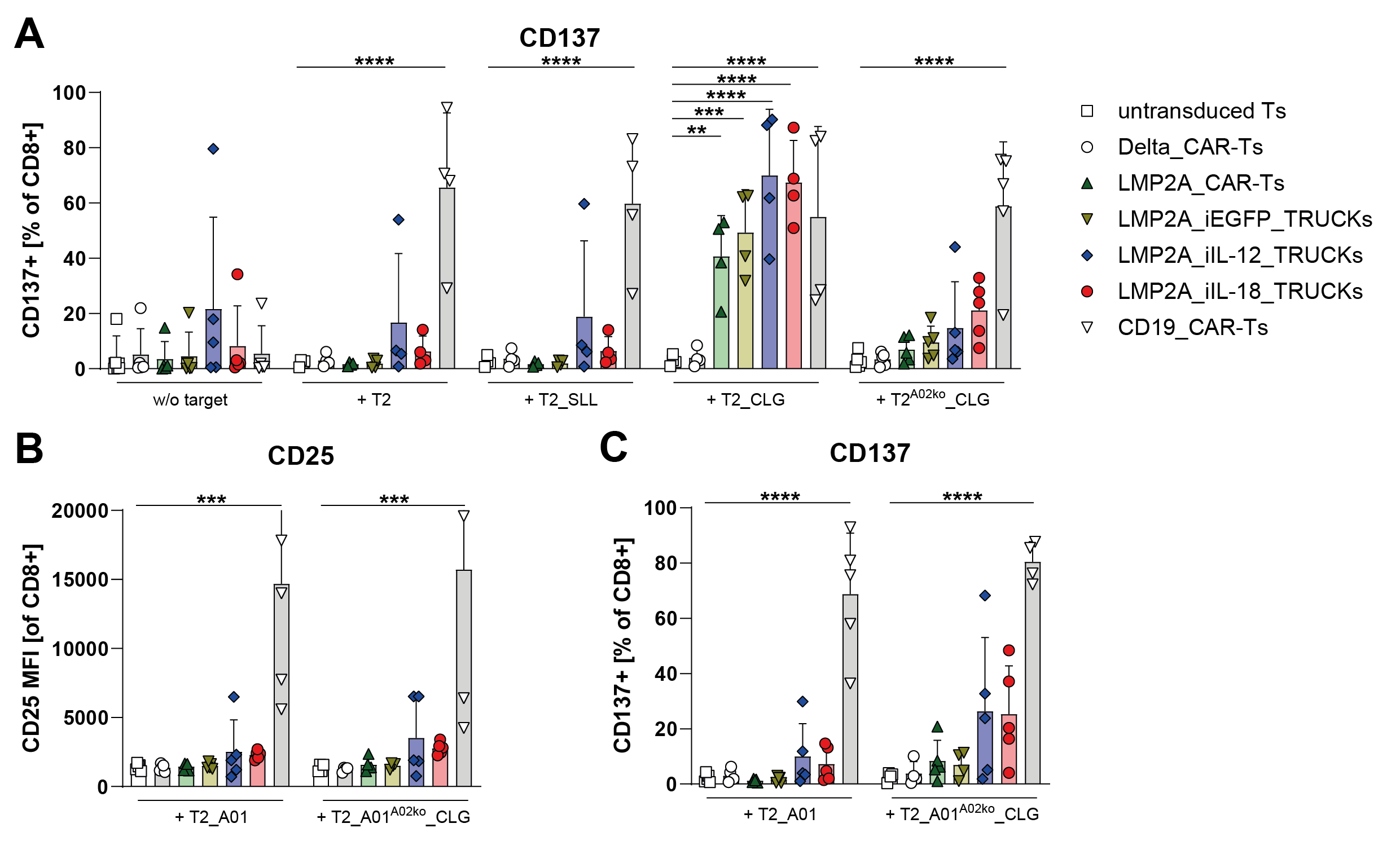


**Figure S4:** LMP2A_CAR‐Ts and LMP2A_TRUCKs are specifically activated following recognition of A02_CLG^+^ T2 cells. LMP2A_CAR‐Ts and LMP2A_TRUCKs were either cultured alone (w/o target) or co‐cultured with T2 cells, T2 cells loaded with the LMP2A‐derived peptide CLGGLLTMV (T2_CLG), T2 cells loaded with the PRAME‐derived control peptide SLLQHLIGL (T2_SLL) or CLG‐loaded T2 cells with *HLA‐A*02:01* knockout (T2^A02ko^_CLG), T2 cells transduced with *HLA‐A*01:01* (T2_A01) or CLG‐loaded T2_A01 with *HLA‐A*02:01* knockout (T2_A01^A02ko^_CLG) for 48 h in an E:T ratio of 1:1. Corresponding co‐cultures with Delta_CAR‐Ts lacking the LMP2A‐specific scFv or CD19_CAR‐Ts with a CD19‐targeting scFv served as controls. Markers for T‐cell activation were determined by flow cytometry and are shown as (A, C) frequency of positive cells or (B) mean fluorescence intensity (MFI). Data are shown as mean + SD, whereby each point represents individual experiment (*n* = 3–5 with T cells from *n* = 3–4 donors). Statistical analysis was performed using two‐way ANOVA and Tukey's multiple comparisons test. **p* ≤ 0.05, ***p* ≤ 0.01, ****p* ≤ 0.001, *****p* ≤ 0.0001.


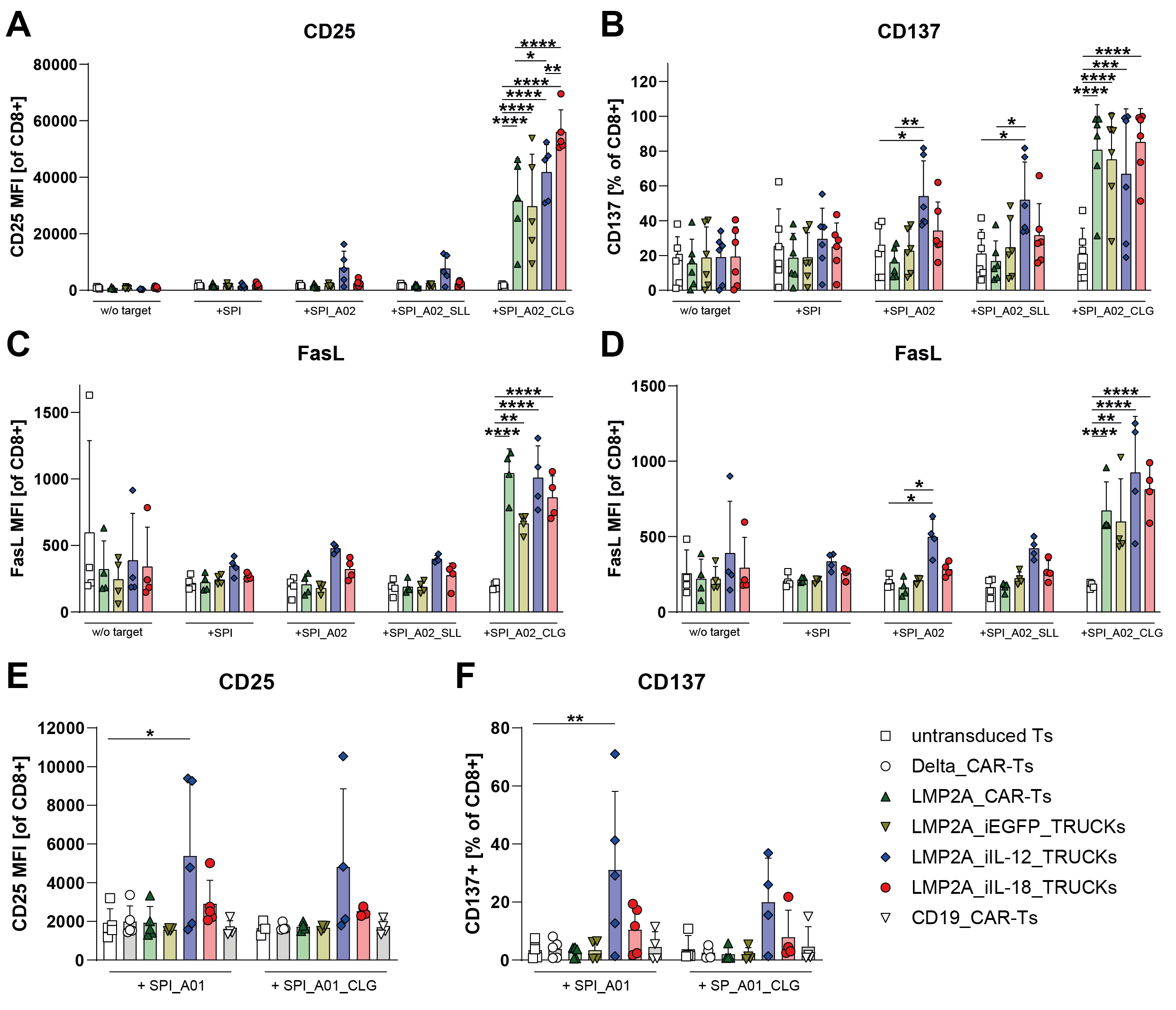


**Figure S5:** LMP2A_CAR‐Ts and LMP2A_iIL‐18_TRUCKs are specifically activated following recognition of A02_CLG^+^ SPI cells, whereas LMP2A_iIL‐12_TRUCKs also respond to A02^+^ or A01^+^ target cells. LMP2A_CAR‐Ts and LMP2A_TRUCKs were either cultured alone (w/o target) or co‐cultured with untransduced SPI‐801 cells (SPI), SPI transduced with *HLA‐A*02:01* either unloaded (SPI_A02), SLL‐loaded (SPI_A02_SLL), CLG‐loaded (SPI_A02_CLG), SPI transduced with *HLA‐A*01:01* (SPI_A01) or CLG‐loaded SPI_A01 (SPI_A01_CLG) for 48 h in an E:T ratio of (A, B, D‐F) 1:1 or (C) 0.5:1. (E, F) Corresponding co‐cultures with Delta_CAR‐Ts lacking the LMP2A‐specific scFv or CD19_CAR‐Ts with a CD19‐targeting scFv served as controls. (A–F) Markers for T‐cell activation were determined by flow cytometry and are shown as (A, C–E) mean fluorescence intensity (MFI) or (B, F) frequency of positive cells. Data are shown as mean + SD, whereby each point represents individual experiment ((A–D) *n* = 4–6 donors, (E, F) *n* = 3–5 with T cells from *n* = 3–4 donors). Statistical analysis was performed using two‐way ANOVA and Tukey's multiple comparisons test. **p* ≤ 0.05, ***p* ≤ 0.01, ****p* ≤ 0.001, *****p* ≤ 0.0001.


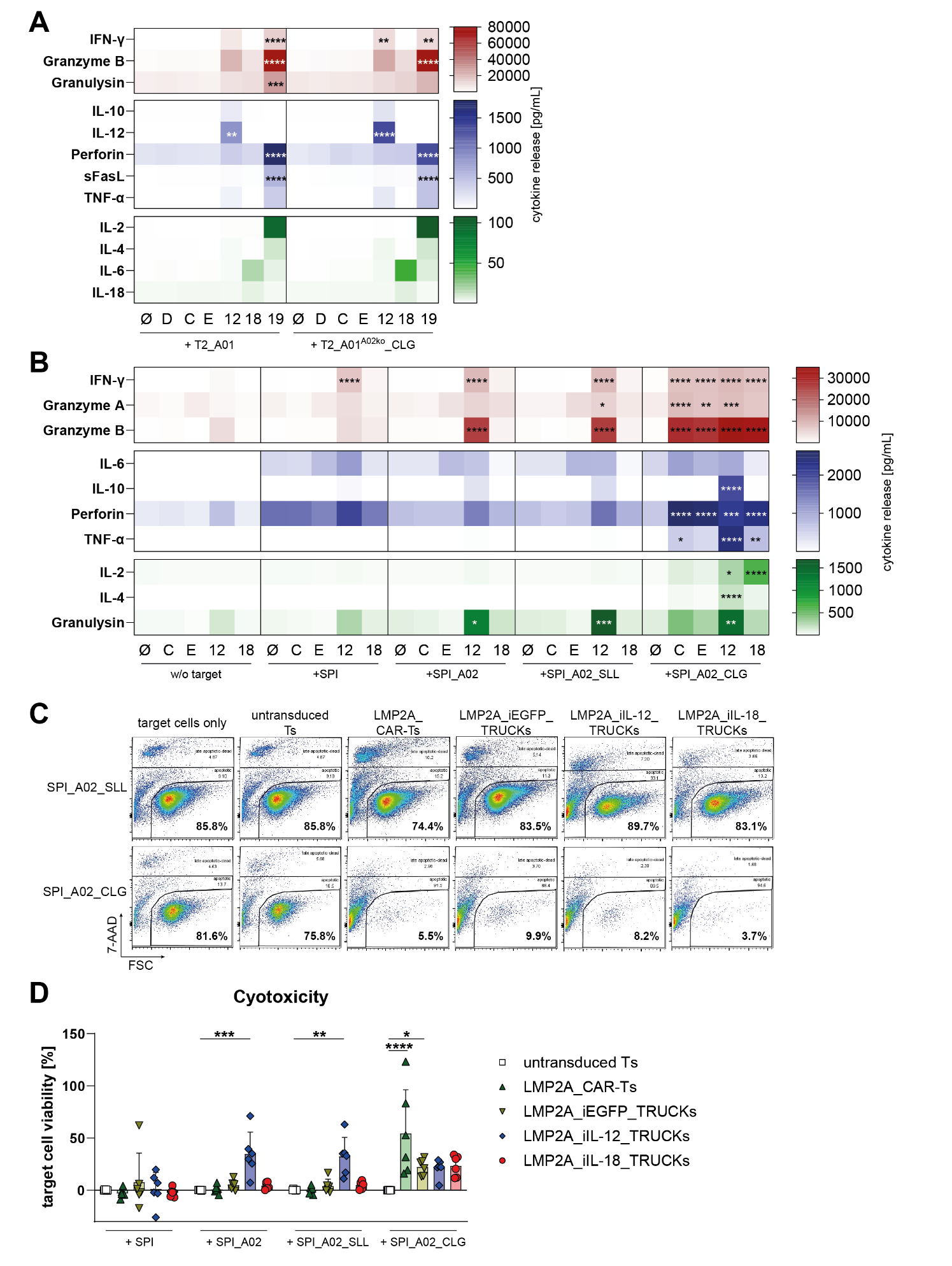


**Figure S6:** LMP2A_CAR‐Ts and LMP2A_iIL‐18_TRUCKs release pro‐inflammatory mediators following recognition of A02_CLG^+^ target cells, whereas LMP2A_iIL‐12_TRUCKs also respond to A02^+^ target cells. LMP2A_CAR‐Ts and LMP2A_TRUCKs were either cultured alone (w/o target) or co‐cultured with (A) T2 cells transduced with *HLA‐A*01:01* (T2_A01), or CLG‐loaded T2_A01 with *HLA‐A*02:01* knockout (T2_A01^A02ko^_CLG) or (B–D) untransduced SPI‐801 cells (SPI), SPI transduced with *HLA‐A*02:01* either unloaded (SPI_A02), CLG‐loaded (SPI_A02_CLG) or SLL‐loaded (SPI_A02_SLL) for 48 h in an E:T ratio of (A, B, D) 1:1 or (C) 0.5:1. (A) Corresponding co‐cultures with Delta_CAR‐Ts lacking the LMP2A‐specific scFv or CD19_CAR‐Ts with a CD19‐targeting scFv served as controls. (A, B) The concentration of different mediators in the culture supernatants was analysed by a bead‐based multiplex cytokine profiling using flow cytometry. Data are shown as mean ((A) *n* = 3–5 with T cells from *n* = 3–4 donors, (B) *n* = 6–8 donors). Statistical analysis was performed using two‐way ANOVA and Tukey's multiple comparisons test. Significant differences to values obtained for untransduced Ts co‐cultured with the same target cells are indicated. 12 = LMP2A_iIL‐12_TRUCKs, 18 = LMP2A_iIL‐18_TRUCKs, 19 = CD19_CAR‐Ts, C = LMP2A_CAR‐Ts, D = Delta_CAR‐Ts, E = LMP2A_iEGFP_TRUCKs, *ø* = untransduced Ts. (C) The cytotoxic activity of LMP2A_CAR‐Ts and LMP2A_TRUCKs was determined by analysing the viability of CTV‐labelled target cells by 7‐AAD staining and subsequent flow cytometry analysis. Data are shown as representative plots for the gating of viable target cells. (D). Cell death in co‐cultures was confirmed by analysis of LDH release into the co‐culture supernatant. LDH levels are expressed in % of the maximum lysis level obtained using controls lysed with 1% Triton X‐100. Data are shown as mean + SD, whereby each point represents one donor (*n* = 6). Statistical analysis was performed using two‐way ANOVA and Tukey's multiple comparisons test. Only significant differences to untransduced T cells co‐cultured with the same target cells are shown. **p* ≤ 0.05, ***p* ≤ 0.01, ****p* ≤ 0.001, *****p* ≤ 0.0001.

**
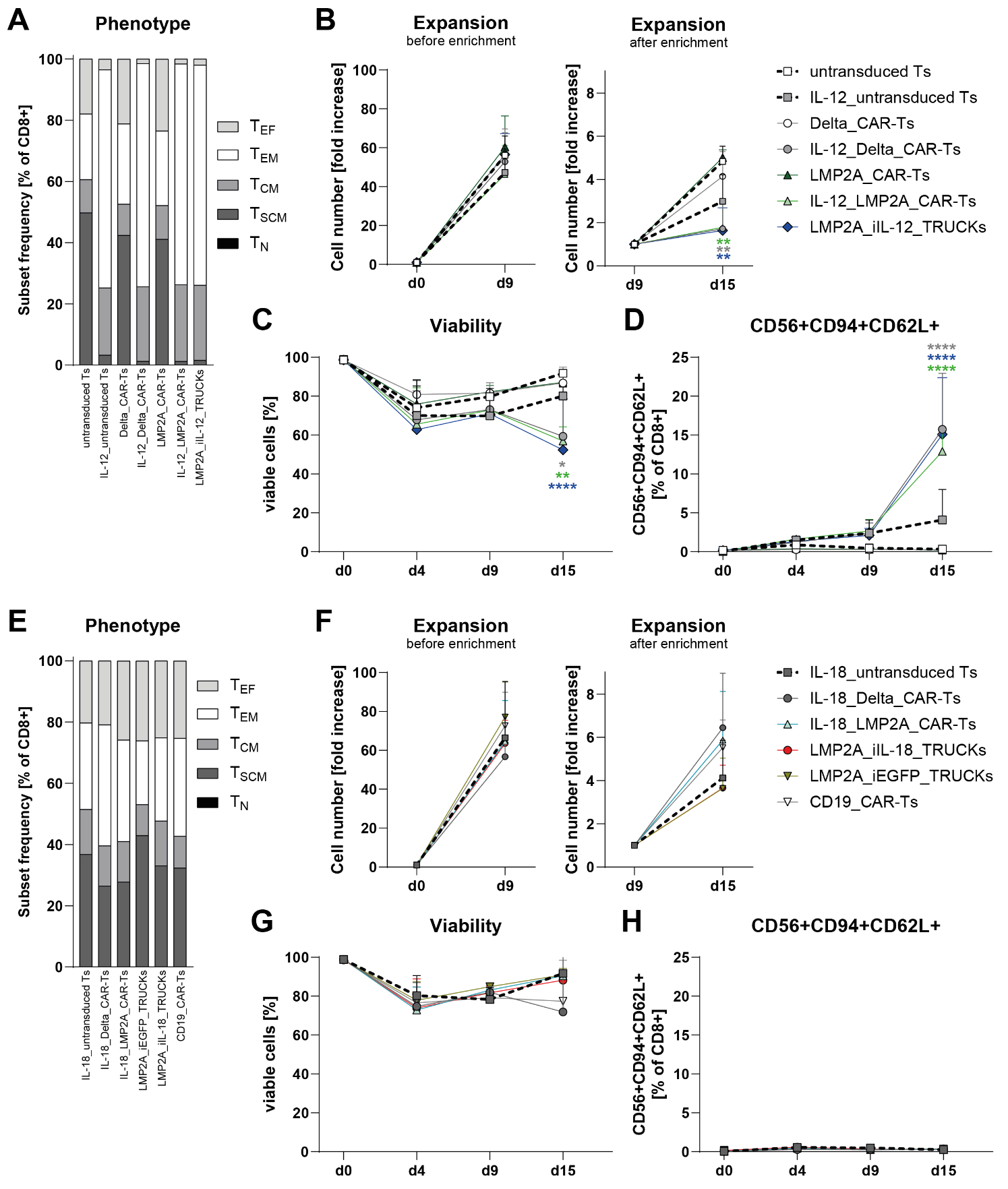
**

**Figure S7:** IL‐12 pre‐conditioning of T cells drives them towards T_EM_ and an NK‐like phenotype that is similar to LMP2A_iIL‐12_TRUCKs. All constructs were transduced into CD8^+^ T cells isolated from healthy individuals. Respective untransduced T cells served as control. To evaluate impact of IL‐12 or IL‐18 pre‐conditioning, these cytokines were exogenously added to untransduced T cells (IL‐12_ or IL‐18_untransduced Ts), Delta_CAR‐Ts (IL‐12_ or IL‐18_Delta_CAR‐Ts) and LMP2A_CAR‐Ts (IL‐12_ or IL‐18_LMP2A_CAR‐Ts) during generation. (A, E) T‐cell memory phenotype was assessed on Day 15 as naïve (T_N_: CD45RO^−^ CCR7^+^ CD95^−^), stem‐cell memory (T_SCM_: CD45RO^−^ CCR7^+^ CD95^+^), central memory (T_CM_: CD45RO^+^ CCR7^+^), effector memory (T_EM_: CD45RO^+^ CCR7^−^) and effector (T_EF_: CD45RO^−^ CCR7^−^) T cells. Data are shown as mean. (B, F) T‐cell expansion was determined by cell counting and is shown as fold increase relative to the respective cell numbers on Day 0 (left) or on Day 9 after enrichment of transduced cells (right). (B, G) Viability of T cells as assessed by 7‐AAD staining, as well as (D, H) frequency of cells with an NK‐like phenotype (CD56^+^CD94^+^CD62L^+^) was assessed on the indicated days using flow cytometry. (B–D, F–H) Data are shown as mean + SD (*n* = 3–4). Statistical analysis was performed using two‐way ANOVA and Tukey's multiple comparisons test. Significant differences to values obtained for (B, F) untransduced Ts or (C, D, G, H) corresponding cells not treated with IL‐12 or IL‐18 at the same day are indicated. **p* ≤ 0.05, ***p* ≤ 0.01, *****p* ≤ 0.0001. D = day.


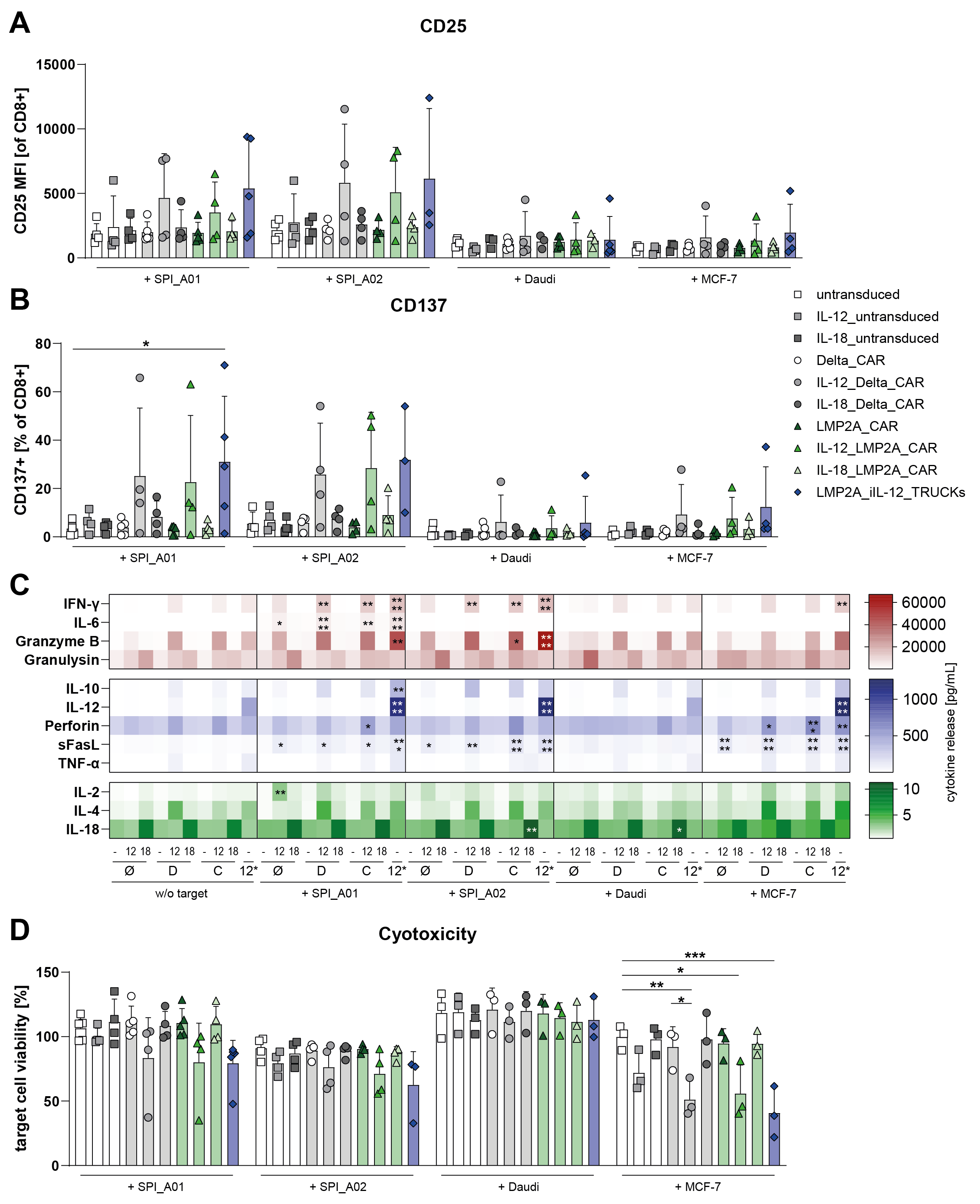


**Figure S8:** IL‐12 pre‐conditioning of transduced T cells induces reactivity towards SPI_A01, SPI_A02 and MCF‐7 cells that is similar to LMP2A_iIL‐12_TRUCKs. To evaluate impact of IL‐12 or IL‐18 pre‐conditioning, these cytokines were exogenously added to untransduced T cells (IL‐12_ or IL‐18_untransduced Ts), Delta_CAR‐Ts (IL‐12_ or IL‐18_Delta_CAR‐Ts) and LMP2A_CAR‐Ts (IL‐12_ or IL‐18_LMP2A_CAR‐Ts) during generation from CD8^+^ T cells isolated from healthy individuals. Their target response was compared to LMP2A_iIL‐12_TRUCKs. All T cells were either cultured alone (w/o target) or co‐cultured with SPI transduced with *HLA‐A*01:01* (SPI_A01) or *HLA‐A*02:01* (SPI_A02), Daudi or MCF‐7 cells for 48 h in an E:T ratio of 1:1. (A, B) Markers for T‐cell activation were determined by flow cytometry and are shown as (A) mean fluorescence intensity (MFI) or (B) frequency of positive cells. (C) The concentration of different mediators in the culture supernatants was analysed by a bead‐based multiplex cytokine profiling using flow cytometry. Significant differences to values obtained for untransduced Ts co‐cultured with the same target cells are indicated. For untransduced Ts (*ø*), Delta_CAR‐Ts (D) and LMP2A_CAR‐Ts (C), conditions without cytokine supplementation (−), with IL‐12 pre‐conditioning (12) or IL‐18 pre‐conditioning (18) were compared. LMP2A_iIL‐12_TRUCKs (12*) were not pre‐conditioned. (D, E) The cytotoxic activity of transduced T cells was determined by analysing the viability of CTV‐labelled target cells by 7‐AAD staining and subsequent flow cytometry analysis. Target cell viability was normalised to viabilities of corresponding target cells cultured alone. Data are shown as (A, B, D) mean + SD or (C) mean, whereby each point represents one individual experiment (*n* = 3–5 with T cells from *n* = 3–4 donors). Statistical analysis was performed using two‐way ANOVA and Tukey's multiple comparisons test. Only significant differences to untransduced T cells and corresponding cells not treated with IL‐12 or IL‐18 when co‐cultured with the same target cells are indicated. (A, B, D) For SPI‐A01 cells, data from co‐cultures with untransduced T cells, Delta_CAR‐Ts, LMP2A_CAR‐Ts and LMP2A_iIL‐12‐TRUCKs are the same as in Figures  and , as those were obtained in the same experiments and are repeated here for comparison with the pre‐conditioned counterparts. **p* ≤ 0.05, ***p* ≤ 0.01, ****p* ≤ 0.001, *****p* ≤ 0.0001.

**Supplementary Tables**

**Supplementary Table S1:** Utilised cell lines and cultivation. RPMI 1640 medium (Lonza), foetal bovine serum (FBS; Merck), L‐glutamine (c.c.pro), DMEM (Lonza).

| **Cell line** | **Order number** | **Supplier** | **Cultivation medium** |
| --- | --- | --- | --- |
| B95-8 | ACC-100 | DSMZ | RPMI 1640, 10% FBS, 2 mM L-glutamine |
| JE6-1 reporter cells |  | Kindly provided by Prof. Peter Steinberger (Medical University of Vienna, Austria) | RPMI 1640, 10% FBS, 2 mM L-glutamine |
| Jurkat | ACC-282 | DSMZ | RPMI 1640, 10% FBS, 2 mM L-glutamine |
| SPI-801 | ACC-86 | DSMZ | RPMI 1640, 10% FBS, 2 mM L-glutamine |
| 293T | ACC-635 | DSMZ | DMEM, 10% FBS, 2 mM L-glutamine |
| T2 | ACC-598 | DSMZ | RPMI 1640, 10% FBS, 2 mM L-glutamine |
| Daudi |  | Kindly provided by Prof. Martin Sauer (Hannover Medical School, Germany) | RPMI 1640, 10% FBS, 2 mM L-glutamine |
| MCF-7 | ACC-115 | DSMZ | RPMI 1640, 10% FBS, 2 mM L-glutamine |

**Supplementary Table S2:** Antibodies used for flow cytometry. Peridinin‐chlorophyll‐protein (PerCP), Alexa Fluor (AF), allophycocyanin (APC), Brilliant Violet (BV), phycoerythrin (PE), fluorescein isothiocyanate (FITC).

| **Antigen** | **Antibody clone** | **Fluorophore** | **Supplier** |
| --- | --- | --- | --- |
| CD137 | 4B4-1 | APC | BioLegend |
| CD178 (FasL) | NOK-1 | PE-Cy7 | BioLegend |
| CD197 (CCR7) | G043H7 | AF700 | BioLegend |
| CD244 (2B4) | C1.7 | AF700 | BioLegend |
| CD25 | BC96 | PE-Cy7 / APC-Cy7 | BioLegend |
| CD3 | SK7 / UCHT1 | FITC / PerCP / AF700 / BV605 | BioLegend |
| CD39 | A1 | APC-Cy7 | BioLegend |
| CD4 | RPA-T4 | BV510 | BioLegend |
| CD45RA | HI100 | PE-Cy7 | BioLegend |
| CD45RO | UCHL1 | APC-Cy7 | BioLegend |
| CD56 | 5.1H11 | BV421 | BioLegend |
| CD62L | DREG-56 | FITC | BioLegend |
| CD62L | DREG-56 | BV510 | BioLegend |
| CD69 | FN50 | BV605 | BioLegend |
| CD8 | SK1 | AF700 / BV510 / APC | BioLegend |
| CD94 | DX22 | FITC | BioLegend |
| CD95 (Fas) | DX2 | BV421 / PE-Cy7 | BioLegend |
| EGFRt | - | biotin | ImClone Systems |
| G4S Linker | E7O2V | biotin | Cell Signaling Technology |
| HLA-A*02:01 | BB7.2 | PE / FITC | BioLegend |
| HLA-ABC | W6/32 | PE | Bio-Rad AbD Serotec |
| LAG-3 | 11C3C65 | BV421 | BioLegend |
| PD-1 | EH12.2H7 | BV605 | BioLegend |
| Streptavidin | - | PE | Thermo Fisher Scientific |
| TIM-3 | F38-2E2 | APC / APC-Cy7 | BioLegend |
